# Efficient and Specific PDGFRβ‐Targeting Dual‐Mode T_1_‐T_2_ MRI Nanoprobe for Early Diagnosis of Non‐Alcoholic Fatty Liver

**DOI:** 10.1002/advs.202413788

**Published:** 2025-03-07

**Authors:** Zehua Li, Tongwei Zhang, Tongxiang Tao, Yaxuan Liu, Haining Xia, Sajid ur Rehman, Zeyong Guo, Jing Zhang, Ruiguo Chen, Zhan Zhang, Junfeng Wang, Kun Ma

**Affiliations:** ^1^ High Magnetic Field Laboratory Key Laboratory of High Magnetic Field and Ion Beam Physical Biology Hefei Institutes of Physical Science Chinese Academy of Sciences Hefei Anhui 230031 P. R. China; ^2^ University of Science and Technology of China Hefei Anhui 230036 P. R. China; ^3^ Key Laboratory of Earth and Planetary Physics Institute of Geology and Geophysics Chinese Academy of Sciences Beijing 100029 P. R. China; ^4^ College of Pharmacy Anhui University of Chinese Medicine 350 Long zi hu Road Hefei 230012 P. R. China; ^5^ Institute of Energy Hefei Comprehensive National Science Center Anhui Energy Laboratory Hefei 230031 P. R. China

**Keywords:** dual‐mode magnetic resonance imaging, early‐stage fibrosis, nanoprobe, PDGFRβ, protein nanocage

## Abstract

Non‐alcoholic fatty liver disease (NAFLD)‐induced early‐stage liver fibrosis is increasingly common. Non‐invasive MRI detection offers an important diagnostic method to prevent fibrosis from progressing to cirrhosis or hepatocellular carcinoma. However, because fibrosis is confined to the periportal areas, and changes in tissue structure and stiffness are minimal, standard T_1_‐ or T_2_‐weighted imaging struggles to capture these early‐stage lesions. To address this challenge, a highly sensitive and targeted T_1_‐T_2_ dual‐mode magnetic resonance imaging (MRI) nanoprobe is designed and developed, specifically targeting early‐stage liver fibrosis characterized by the activation of hepatic stellate cells (HSCs) and the overexpression of platelet‐derived growth factor receptor β (PDGFRβ). The nanoprobe exhibits excellent relaxivity (r_2_/r_1_ = 10.7) and precise targeting due to PDGFRβ‐specific peptides conjugated to its protein nanocage. In vivo, imaging in animal models demonstrate effective accumulation of the probe in fibrotic regions as NAFLD progressed, with fluorescence signal intensity accurately reflecting the severity of liver fibrosis. Using a 7T MRI system, T_1_ and T_2_ images are overlaid within 1 h, accurately locating fibrotic areas and improving diagnostic speed and precision. The nanoprobe shows excellent biocompatibility and enhances early fibrosis detection in NAFLD, offering significant clinical potential for early diagnosis, prognosis, and recurrence monitoring.

## Graphical abstract

The Fe_3_O_4_/Gd@BSA‐pPB nanoprobe leverages biomimetic mineralization and targets hepatic stellate cells (HSCs) through PDGFRβ, offering superior enrichment in fibrotic regions. This targeted delivery overcomes biological barriers, enhancing both the accuracy and sensitivity of MRI imaging at early disease stages. Through dual T_1_ and T_2_ imaging, this nanoprobe provides complementary signal enhancement, improving contrast between fibrotic and healthy liver tissue, while minimizing image artifacts.

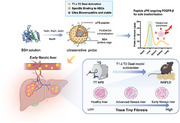

## 1 Introduction

Non‐alcoholic fatty liver disease (NAFLD) is a metabolic disorder characterized by fat accumulation exceeding 5–10% of liver weight in individuals without significant alcohol consumption.^[^ ^1^ ^]^ Its hallmark is diffuse, large‐droplet fatty degeneration within the liver. Globally, NAFLD affects ≈25–30% of adults, with prevalence increasing to 60–90% in individuals with obesity or type 2 diabetes.^[^ ^2^, ^3^, ^4^ ^]^ Without timely intervention, NAFLD can progress to non‐alcoholic steatohepatitis (NASH), triggering lipotoxicity, endoplasmic reticulum stress, and inflammation.^[^ ^5^, ^6^ ^]^ Over half of affected individuals may develop liver fibrosis, which can further progress to cirrhosis or hepatocellular carcinoma (HCC).^[^ ^7^, ^8^, ^9^ ^]^ Liver fibrosis refers to the abnormal accumulation of fibrous tissue, primarily collagen, in response to chronic liver injury, though it has not yet reached the cirrhotic stage. Early detection and management of fibrosis can slow or even reverse the progression of NAFLD.^[^ ^10^ ^]^ Despite the presence of mild fibrosis, liver function typically remains intact, with only slight elevations in liver enzymes (ALT and AST) or levels within the normal range.^[^ ^11^, ^12^ ^]^ Currently, invasive liver biopsy remains the most accurate method for diagnosing early to mid‐stage fibrosis, although it poses risks of significant discomfort and potential complications.^[^ ^13^ ^]^

Recent advancements in non‐invasive MRI technology have enabled high‐resolution imaging of liver structures, proving particularly valuable for detecting early and intermediate stages of fibrosis by revealing morphological changes in fibrotic areas.^[^ ^14^, ^15^ ^]^ However, in the early stages of fibrosis, the pathological changes are often confined to periportal areas, resulting in minimal alterations in overall liver structure, stiffness, or water and fat content.^[^ ^16^ ^]^ Consequently, the contrast in MRI signals may be insufficient to detect these subtle lesions in standard T_1_‐ or T_2_‐weighted imaging.^[^ ^17^ ^]^ Moreover, during early and intermediate fibrosis, the accumulation of extracellular matrix (ECM) and collagen fibers is often not substantial enough to cause significant signal changes, making it difficult to distinguish fibrotic areas from normal liver tissue on MRI.^[^ ^18^ ^]^ The liver's deep anatomical location, surrounded by complex structures such as the intestines, bile ducts, and blood vessels, can further introduce artifacts and signal noise in MRI images, compromising image quality. This is especially problematic in early‐stage fibrosis, where small signal changes may be obscured by such artifacts. MRI probes for liver disease diagnosis can be broadly classified into two main categories. The first includes probes based on traditional Gd and Mn contrast agents, such as small‐molecule and macromolecular derivatives of Gd ion chelates, including those specifically targeting liver type I collagen. These probes demonstrate notable specificity.^[^ ^19^ ^]^ The second category comprises probes based on inorganic nanomaterials, such as NaGdF4, GdO, and MnO, which significantly enhance longitudinal relaxivity and exhibit unique environmental responsiveness. These properties enable superior contrast enhancement in MRI imaging compared to conventional Gd‐DTPA agents.^[^ ^20^, ^21^ ^]^ Despite these advancements, several challenges remain, such as safety concerns, including the need to reduce Gd ion leakage and enhance renal metabolism of inorganic nanomaterials. Additionally, improving target specificity is crucial, as some probes struggle to traverse complex biological barriers, resulting in reduced imaging accuracy.^[^ ^22^ ^]^ Therefore, the development of a highly sensitive MRI probe is essential to enhance the sensitivity and specificity of liver fibrosis diagnosis.^[^ ^23^ ^]^

Hepatic stellate cells (HSCs) play a pivotal role in the process of liver fibrosis.^[^ ^24^ ^]^ Under normal conditions, HSCs remain in a quiescent state between hepatic sinusoids and hepatocytes. Upon liver injury, HSCs become activated and express high levels of platelet‐derived growth factor receptor‐beta (PDGFRβ).^[^ ^25^ ^]^ The PDGF family (PDGF‐A to PDGF‐D), by binding to PDGFRβ, promotes HSC proliferation and migration, which in turn drives collagen deposition and the progression of fibrosis.^[^ ^26^ ^]^ In recent years, various pPB cyclic peptides have been developed to specifically recognize and bind to HSCs, minimizing non‐specific effects on other liver cells.^[^ ^27^ ^]^ Therefore, imaging techniques designed to target PDGFRβ on HSCs can detect pathological changes associated with fibrosis at an earlier stage, rather than relying solely on tissue stiffness or structural changes. This significantly enhances the sensitivity of MRI for early fibrosis diagnosis.^[^ ^28^ ^]^

**Scheme 1:**
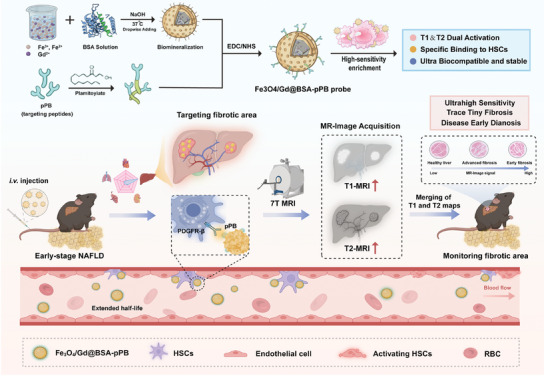
Schematic illustration of the ultrasensitive Fe_3_O_4_/Gd@BSA‐pPB probe for the early diagnosis of liver fibrosis in NAFLD using T_1_ and T_2_ dual‐mode imaging.

Compared to single‐mode contrast agents, which utilize either T_1_ or T_2_ weighting, the combination of both T_1_ and T_2_ modalities offers superior visualization of target tissues, such as fibrotic regions.^[^ ^29^ ^]^ T_1_ agents increase signal intensity (brightness), making fibrotic areas more distinct in images, while T_2_ agents reduce signal intensity from surrounding tissues, thereby enhancing the contrast between the fibrotic region and adjacent normal tissue.^[^ ^30^, ^31^ ^]^ This dual‐mode approach overcomes the limitations of single‐mode agents, particularly in the detection of early‐stage lesions. The complementary signals from T_1_ and T_2_ reduce the likelihood of misinterpretation due to artifacts or noise.^[^ ^32^ ^]^ If the given area shows abnormalities in both T_1_‐ and T_2_‐weighted images, the region is more likely to contain pathological changes, especially in the context of early‐stage fibrosis. In light of these advantages, the development of dual‐mode T_1_/T_2_ MRI probes targeting HSCs presents a promising solution for addressing the limitations of traditional MRI in detecting early and intermediate‐stage liver fibrosis. Here, recognizing the early activation of HSCs and the overexpression of PDGFRβ receptors during the progression of NAFLD, we have designed and developed a highly sensitive and specific Fe_3_O_4_/Gd@BSA‐pPB dual‐mode T_1_‐T_2_ MRI nanoprobe through biomimetic mineralization (As shown in the schematic diagram). This nanoprobe demonstrates excellent r_1_ and r_2_ relaxivity values, exhibiting the typical characteristics of dual‐mode imaging, thereby significantly enhancing the detection capabilities for early‐stage fibrosis. Additionally, through the efficient conjugation of antibody fragments that specifically bind to PDGFRβ, the dual‐mode nanoprobe is effectively enriched in fibrotic regions as NAFLD progresses. These metabolizable and biocompatible nanoprobes are capable of generating high‐resolution MRI images, accurately depicting the progression of fibrosis at different stages while minimizing artifact interference and enhancing diagnostic reliability (**Scheme** **1**).

## 2 Results and Discussion

### 2.1 Characterization of Fe_3_O_4_/Gd@BSA Nanoparticles

**Figure 1:**
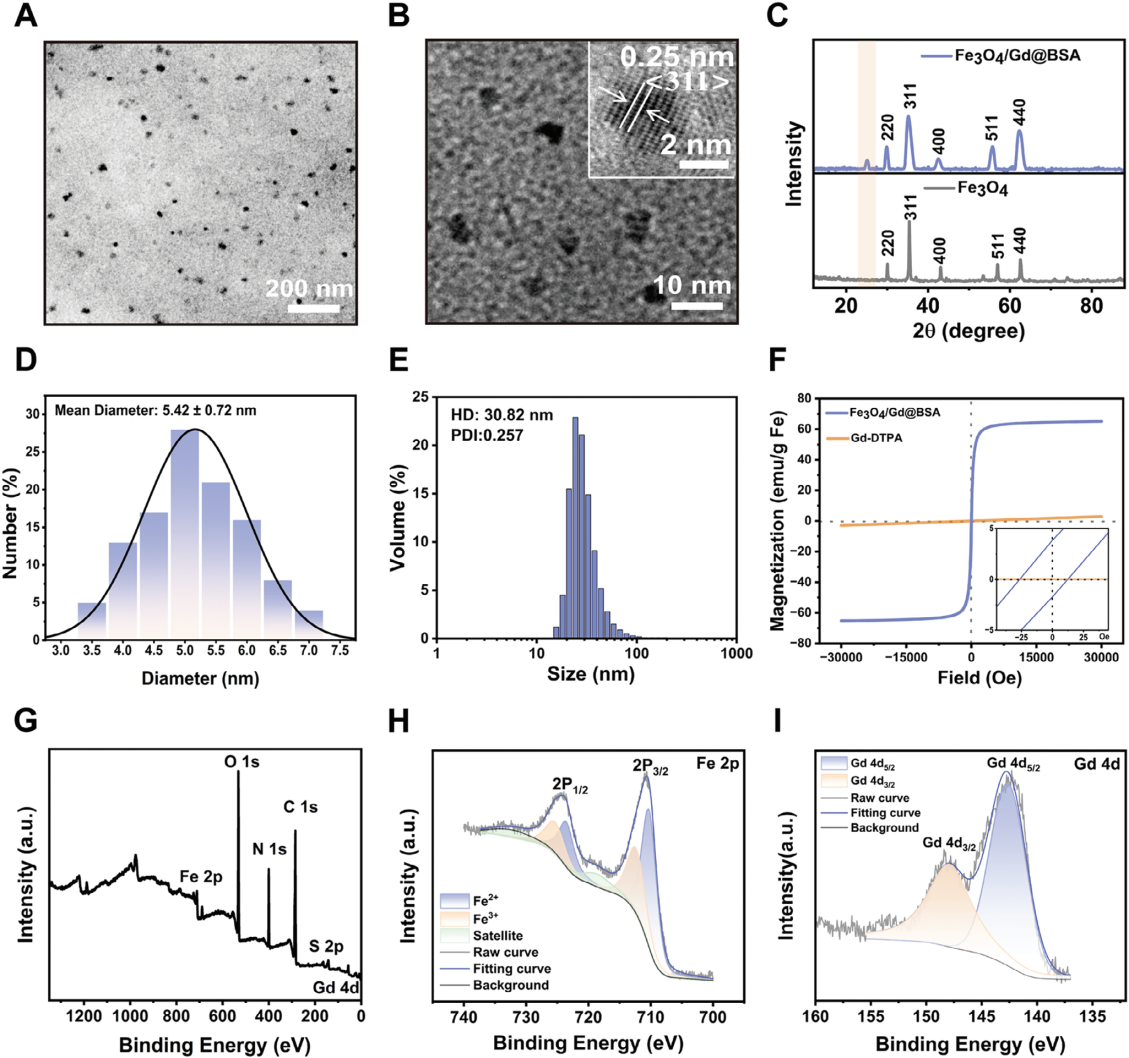
Characterization of Fe_3_O_4_/Gd@BSA nanoparticles. The TEM images A) and Lattice of HRTEM image B) of Fe_3_O_4_@BSA nanoparticles. C) XRD pattern of Fe_3_O_4_/Gd@BSA nanoparticles and Fe_3_O_4_. The size distribution D) and Hydrodynamic diameter E) of Fe_3_O_4_/Gd@BSA nanoparticles. F) Field‐dependent magnetization curves (*M*–*H*) and coercive force (*Hc*) at 300 K. G) Full‐scan XPS spectrum of Fe_3_O_4_/Gd@BSA. High‐resolution XPS spectrum of Fe 2p H) and Gd 4d I).

The currently reported processes for preparing small‐sized iron oxide nanoparticles primarily include high‐temperature thermal decomposition and hydrothermal techniques, which are often cumbersome and time‐consuming.^[^ ^33^, ^34^ ^]^ Additionally, most nanoparticles are synthesized in the oil phase, rendering them insoluble in water.^[^ ^35^ ^]^ Consequently, the synthesized particles need to be replaced with hydrophilic ligands, this process undoubtedly increases the difficulty of the synthesis again while decreasing the yield.^[^ ^36^ ^]^ Protein molecules have the advantages of high biocompatibility, biodegradability, and low toxicity.^[^ ^37^ ^]^ We prepared Fe_3_O_4_/Gd@BSA nanoparticles based on BSA‐mediated biomimetic mineralization synthesis, which is a method with simple reaction conditions, scalability, and high clinical translational potential.^[^ ^38^, ^39^ ^]^ Transmission electron microscopy (TEM) image indicated that by varying ion ratios and optimizing BSA concentration, we successfully synthesized Fe_3_O_4_/Gd@BSA nanoparticles with a particle size of 5.42 ± 0.72 nm (**Figure** **1**A,D). High‐resolution TEM (HRTEM) provided additional details about the Fe_3_O_4_/Gd@BSA nanoparticles, confirming their crystalline nature (Figure 1B). The calculated lattice spacing was 0.25 nm, consistent with the 311 crystal plane of the Fe_3_O_4_ cubic structure (Figure S1, Supporting Information). The X‐ray diffraction (XRD) pattern of Fe_3_O_4_/Gd@BSA is depicted in Figure 1C, where the relative intensities of the diffraction peaks were observed to match the standard JCPDS data for Fe_3_O_4_ (JCPDS No. 89–3854). Based on the TEM data, the broad peak at 2*θ* = 23° of the average grain diameter was estimated to be the crystallographic structural domain of the BSA protein using Scherrer's formula. This indicates that BSA has been successfully introduced into the nanoparticles. The hydrodynamic diameter measured by dynamic light scattering (DLS) was 30.82 nm, further confirming the homogeneity of the Fe_3_O_4_/Gd@BSA nanoparticles (Figure 1E). Additionally, Gd was successfully loaded into the BSA protein cages, as detected by energy‐dispersive spectroscopy (EDS) and confirmed by energy dispersive X‐ray spectroscopy (EDX) elemental mapping images (Figures S2 and S3, Supporting Information). The magnetic properties of nanoparticles are primarily influenced by their size. As the particle size decreases, the magnetic moment diminishes due to reduced magnetic anisotropy and increased surface spin disorder. This characteristic makes ultra‐small magnetic nanoparticles advantageous for enhancing r_1_. To evaluate the feasibility of Fe_3_O_4_/Gd@BSA nanoparticles as contrast agents, we characterized their magnetic properties using a superconducting quantum interference device (SQUID) magnetometry at 300 K, obtaining magnetic field‐dependent magnetization curves (*M*–*H*). As shown in Figure 1F, the saturation magnetization (*Ms*) of the nanoparticles is 65 emu g^−1^ Fe, while the coercivity (*Hc*) is minimal and nearly negligible, which is due to the magnetic anisotropy induced by the smaller size of the particles. The successful doping of Gd and the chemical states of elements in Fe_3_O_4_/Gd@BSA nanoparticles was assessed by X‐ray photoelectron spectroscopy (XPS). The full‐scan XPS spectra revealed characteristic peaks corresponding to the elements Fe 2p, Gd 4d, and O 1s (Figure 1G; Figure S4, Supporting Information), confirming the presence of oxygen, gadolinium, and iron elements in Fe_3_O_4_/Gd@BSA, thus indicating successful Gd incorporation into the magnetic nanoparticles. The binding energies at 710.4 and 724.3 eV are attributed to Fe 2p 3/2 and Fe 2p 1/2 cations, respectively, confirming the presence of both Fe^3+^ and Fe^2+^ (Figure 1H).^[^ ^40^, ^41^ ^]^ The Gd 4d peaks corresponding to 142.6 and 148.1 eV proved the presence of Gd (Figure 1I).^[^ ^42^ ^]^

### 2.2 Design and Characterization of Ultrasensitive Fe_3_O_4_/Gd@BSA‐pPB Probe

**Figure 2:**
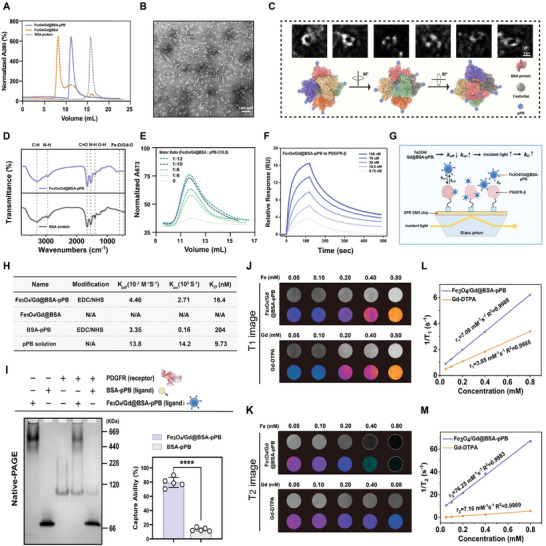
Design and characterization of ultrasensitive Fe_3_O_4_/Gd@BSA‐pPB probe. A) The SEC profiles in Superose 6 Increase 10/300 GL column and Negative staining TEM of Fe_3_O_4_/Gd@BSA‐pPB probe B,C) The size, shape, and main structural features are represented as cartoons. D) FTIR spectra of Fe_3_O_4_/Gd@BSA‐pPB probe and BSA protein. E) Different ratios of Fe_3_O_4_/Gd@BSA and pPB in the click reaction. Affinity curves of Fe_3_O_4_/Gd@BSA‐pPB for PDGFRβ F) and high‐affinity working mode for SPR assays G,H,I) Differences in the ability to capture PDGFRβ in Fe_3_O_4_/Gd@BSA‐pPB probe and BSA‐pPB protein. T_1_ and T_2_ MR imaging J,K) and relaxation rates L,M). The data are shown as the means ± SEM. ^****^ *p* < 0.0001; 2‐tailed Student's *t*‐test (I). Scale bar = 100 nm, Magnified image = 2 nm.

Targeting is a crucial criterion for assessing the effectiveness of a probe.^[^ ^43^, ^44^ ^]^ Using a click reaction, we modified the surface of Fe_3_O_4_/Gd@BSA nanoparticles with the cyclic peptide pPB, which specifically binds to PDGFRβ, a protein found on the surface of hepatic stellate cells (HSCs).^[^ ^45^ ^]^ To obtain a single, homogeneous, and ultrasensitive Fe_3_O_4_/Gd@BSA‐pPB probe, we purified the product from the click reaction via size‐exclusion chromatography (SEC) (**Figure** **2**A; Figure S5, Supporting Information). The purified Fe_3_O_4_/Gd@BSA‐pPB probe was stained with uranyl acetate for negative staining to facilitate TEM image observation. We selected over 10 000 particles from this set of micrographs and performed 2D alignment and classification, iterating several times to converge on the final mean value for each class. A cartoon model was generated through molecular dynamics (MD) simulation and computational modeling (Figure 2C), depicting six BSA subunits closely packed into a spherical complex, with biomineralized Fe_3_O_4_ and Gd nanoparticles encapsulated in the core region of the BSA protein cage. The cyclic peptide pPB, modified via click reaction, was externally coupled, exposing numerous hydrophilic residues on the surface of the Fe_3_O_4_/Gd@BSA‐pPB probe. This modification significantly enhanced the interaction of the nanoprobe with water and improved its stability in aqueous solutions. Figure 2D shows the normalized FTIR spectra of the Fe_3_O_4_/Gd@BSA‐pPB probe and BSA protein, from which the absorption peaks of Fe─O and Gd─O in the range of 570–600 cm^−1^ can be clearly observed.^[^ ^46^, ^47^ ^]^ The remaining peaks corresponding to C─H, N─H, and amide bonding are consistent with those of the BSA protein.^[^ ^48^ ^]^ To enhance the binding affinity of the Fe_3_O_4_/Gd@BSA‐pPB probe to target proteins, we optimized the molar ratio of the protein cage to pPB in the click reaction to 1:10 (Figure 2E; Figure S6, Supporting Information). We assessed the binding of the Fe_3_O_4_/Gd@BSA‐pPB probe to the target protein PDGFRβ across a range of concentrations using surface plasmon resonance (SPR) (Figure 2F). Due to the modification of the Fe_3_O_4_/Gd@BSA protein cage surface with numerous cyclic peptides pPB that specifically bind to PDGFRβ, resulting in a stronger binding affinity for PDGFRβ compared to BSA‐pPB and an elevated affinity (Figure 2G). The affinity of the Fe_3_O_4_/Gd@BSA‐pPB probe for PDGFRβ was measured at 16.4 nm, which is 12.4‐fold higher than that of BSA‐pPB (Figure 2H; Figures S7 and S8, Supporting Information). Furthermore, we captured free PDGFRβ protein in vitro using both the Fe_3_O_4_/Gd@BSA‐pPB probe and BSA‐pPB, and Native‐PAGE analysis revealed that the Fe_3_O_4_/Gd@BSA‐pPB probe had a six‐fold higher capacity to capture PDGFRβ compared to BSA‐pPB in vitro (Figure 2I; Figure S9, Supporting Information). In this work, Gd is biomineralized with Fe_3_O_4_ and is encapsulated into the cage‐like structure formed by BSA to form the Fe_3_O_4_/Gd@BSA‐pPB probe for simultaneous dual T_1_‐ and T_2_‐weighted imaging for early diagnosis of NAFLD fibrosis. A series of Fe_3_O_4_/Gd@BSA‐pPB preparations with varying concentrations were evaluated, and the longitudinal and transverse relaxation rates were investigated using a 3T clinical MRI scanner in vitro. As shown in Figure 2J,K, the MR images clearly showed that the brightening and darkening effects increased with increasing Gd and Fe concentrations, respectively. It confirms that the Fe_3_O_4_/Gd@BSA‐pPB probe has both T_1_‐ and T_2_‐weighted double contrast potentials in MR‐imaging. Additionally, the T_1_ longitudinal and T_2_ transverse relaxation rates were calculated to quantitatively assess the dual T_1_‐ and T_2_‐weighted MR contrast capabilities of the Fe_3_O_4_/Gd@BSA‐pPB probe (Figure 2L,M). Notably, the Fe_3_O_4_/Gd@BSA‐pPB probe exhibited an enhanced r_1_ relaxation rate of 7.08 mm ^−1^s^−1^ compared to the commercial product Gd‐DTPA, which had a rate of 4.45 mm ^−1^ s^−1^, with an r_2_/r_1_ ratio of 10.7, confirming its efficacy as a dual‐mode contrast agent.

### 2.3 Validation of Ultrasensitive Fe_3_O_4_/Gd@BSA‐pPB Probe Binding to Activated Hepatic Fibroblasts In Vitro

**Figure 3:**
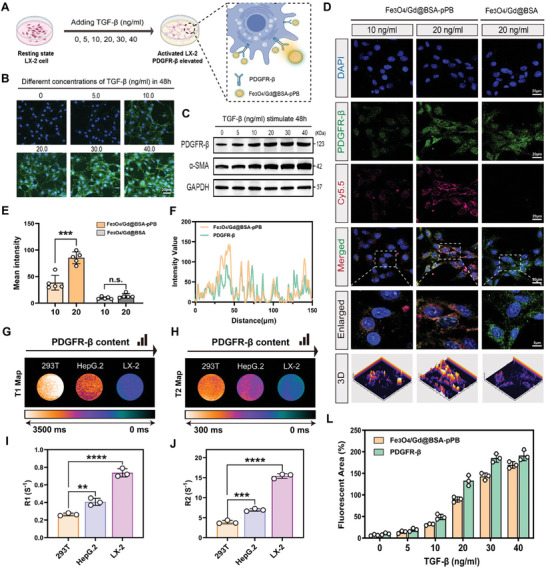
Validation of ultrasensitive Fe_3_O_4_/Gd@BSA‐pPB probe binding to activated hepatic fibroblasts in vitro. A) Schematic representation illustrating the further activation of hepatic fibrosis by TNF‐β stimulation of LX‐2 cells in vitro. B) Different concentrations of TNF‐β were added to LX‐2 cells in the quiescent state, followed by immunofluorescence detection of PDGFβ expression using labeled antibodies after 48h. C) Western blot analysis of relative protein expression of PDGFRβ and α‐SMA in response to stimulation with different concentrations of TNF‐β. D) Dual fluorescence immunofluorescence analysis of Cy5.5‐labeled Fe_3_O_4_/Gd@BSA‐pPB with PDGFRβ. E) Fluorescence intensity of Fe_3_O_4_/Gd@BSA‐pPB and Fe_3_O_4_/Gd@BSA incubations after TNF‐β stimulation (10, 20 ng mL^−1^, respectively). F) The fluorescence co‐localization detection of Fe_3_O_4_/Gd@BSA‐pPB probe with PDGFRβ. G,H) T_1_ Map and T_2_ Map of different cell lines (293T, HepG.2, and LX‐2) incubated with Fe_3_O_4_/Gd@BSA‐pPB probe. Representative data are shown from three independent samples. I,J) R_1_ and R_2_ of different cell lines (293T, HepG.2, and LX‐2) incubated with Fe_3_O_4_/Gd@BSA‐pPB probe. L) Fluorescence intensity analysis of ultrasensitive probes and PDGFRβ under different concentrations of TNF‐β stimulation. The data are shown as the means ± SEM. ^**^ *p* < 0.01, ^***^ *p* < 0.001, ^****^ *p* < 0.0001, n.s.: not statistically significant; 2‐way ANOVA (E, I, J). Scale bar = 20 µm.

The persistent activation of HSCs is pivotal in the formation of hepatic fibrosis, leading to excessive deposition of extracellular matrix (ECM).^[^ ^49^ ^]^ This activation is closely associated with the addition of transforming growth factor (TGF‐β).^[^ ^50^ ^]^ As shown in **Figure** **3**A, different concentrations of TGF‐β were introduced to starvation‐treated quiescent state LX‐2 cells for 48 h to simulate the early stages of hepatic fibrosis. Following stimulation, the LX‐2 cells were subjected to immunofluorescence staining. The results indicated that the expression of PDGFRβ increased in response to TGF‐β stimulation (green) (Figure 3B). Western blot and mRNA level analysis confirmed that the protein expression of PDGFRβ correlated with the elevated levels of α‐SMA, a known marker of fibrosis (Figure 3C; Figure S10, Supporting Information). These findings indicate that PDGFRβ is specifically expressed in early fibroblasts and can effectively trace the progression of early fibrosis. To further assess the targeting capability and sensitivity of the Fe_3_O_4_/Gd@BSA‐pPB probe, we performed cell immunofluorescence detection using Cy5.5‐labeled Fe_3_O_4_/Gd@BSA‐pPB probes, with Cy5.5‐labeled Fe_3_O_4_/Gd@BSA serving as the control. LX‐2 cells in a resting state were incubated with two concentrations of TNF‐β (10 and 20 ng mL^−1^) for 48 h to induce fibrosis. Following this, the cells were co‐cultured with the Cy5.5‐labeled Fe_3_O_4_/Gd@BSA‐pPB probe at 37 °C for 4 h. The results demonstrated that the Fe_3_O_4_/Gd@BSA‐pPB probe exhibited high sensitivity in distinguishing early liver fibrosis cells, with the fluorescent probe signal (Cy5.5, red) co‐localizing with PDGFRβ protein (immunofluorescence staining, green) on the surface of activated LX‐2 cells. Furthermore, the fluorescence intensity of the enriched probe increased with higher PDGFRβ expression, indicating that the Fe_3_O_4_/Gd@BSA‐pPB probe specifically targeted activated HSCs that overexpress PDGFRβ. In contrast, as a control, Fe_3_O_4_/Gd@BSA exhibited only weak fluorescence intensity due to the absence of the targeting protein pPB (Figure 3D–F). To further assess the MRI imaging properties of the ultrasensitive Fe_3_O_4_/Gd@BSA‐pPB probe, three different cell lines‐293T, HepG.2, and activated LX‐2 were selected and incubated with the probe for 4 h. The 293T cell line represents normal cells, HepG.2 represents hepatocellular carcinoma cells, and activated LX‐2 represents hepatic fibrosis cells, all of which showed a gradual increase in PDGFRβ expression. During this period, the ultrasensitive probe exhibited significantly lower toxicity to the cells (Figure S11, Supporting Information). The data presented in Figure 3G,H indicates that both T_1_ and T_2_ images of the 293T group maintained low‐contrast signals. In contrast, as PDGFRβ expression increased in the cells, the corresponding images of the HepG.2 and LX‐2 cell groups exhibited significantly enhanced signal levels. The T_1_‐ and T_2_‐weighted images showed progressively “activated” signals. Additionally, the R_1_ and R_2_ values, calculated from the MRI images (Figure 3I,J), also demonstrated a gradual increase. With the addition of gradient TGF‐β, PDGFRβ protein was elevated and the enrichment signal of the ultrasensitive probe was accompanied by enhanced (Figure 3L). These results confirm that the Fe_3_O_4_/Gd@BSA‐pPB probe effectively binds to elevated PDGFRβ proteins on the surface of early fibrotic cells, highlighting its significant potential for the diagnosis of early fibrosis in vivo.

**Figure 4:**
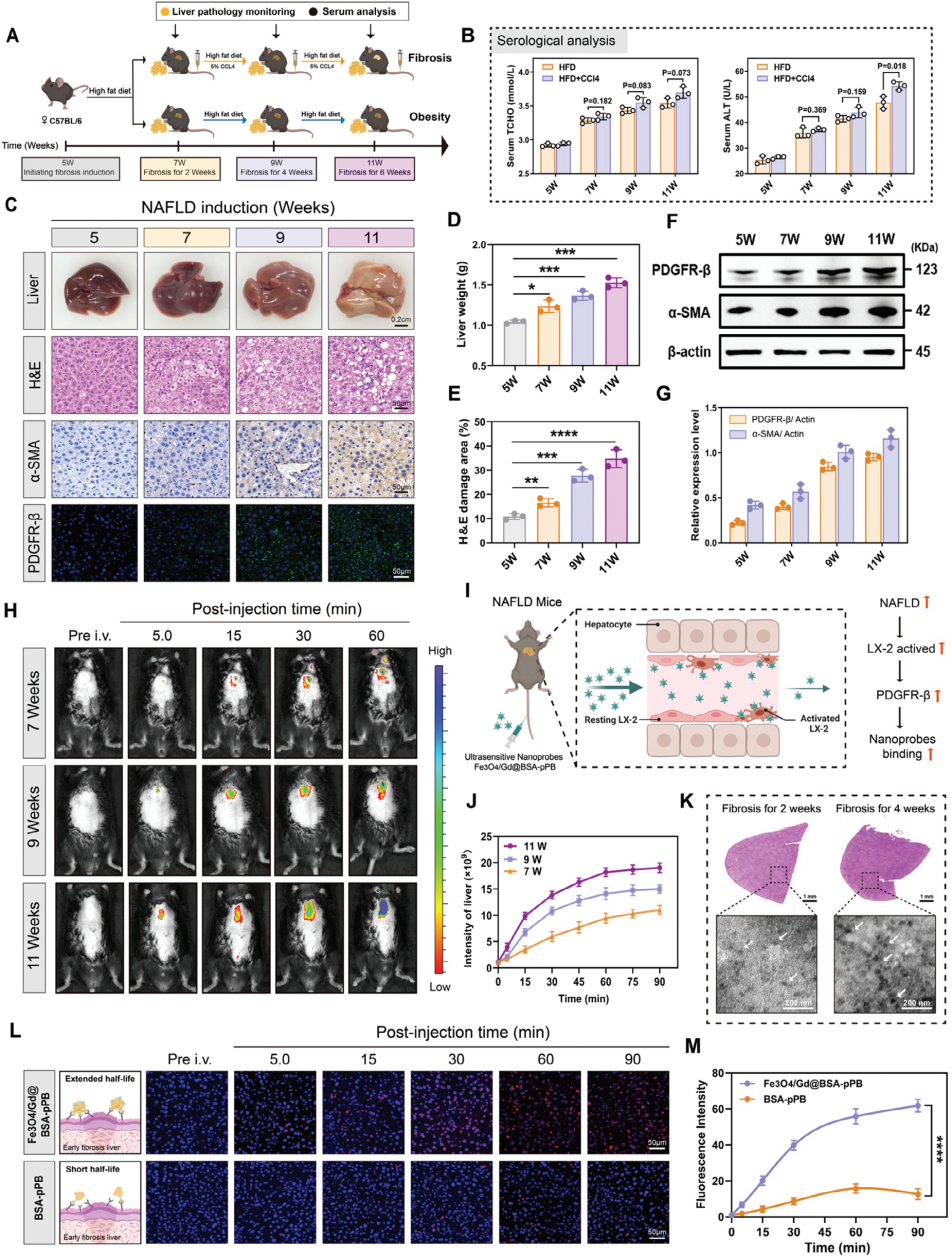
Establishment of early nonalcoholic fatty liver fibrosis and validation of ultrasensitive Fe_3_O_4_/Gd@BSA‐pPB probe targeting in pathological situations. A) Schematic diagram of the high‐fat diet and CCl_4_‐induced early NAFLD fibrosis model in mice (n = 6/group). B) Serological analysis (TCHO, ALT) of early liver fibrosis models for different induction times. Liver phenotype, HE staining, α‐SMA staining and PDGFRβ fluorescent staining C), Liver weight D), HE damages area E) and PDGFRβ, α‐SMA expression F,G) in an early liver fibrosis model with different induction times. H) Representative fluorescence images of early liver fibrosis C57BL/6 mice induced at 7, 9, and 11 weeks after 60min intravenous injection of Fe_3_O_4_/Gd@BSA‐pPB‐Cy5.5 (1 nmol g^−1^ Cy5.5). I) Mechanism diagram of ultrasensitive Fe_3_O_4_/Gd @BSA‐pPB probe in early liver fibrosis. J) Liver ROI intensity at different times after injection. K) TEM images of liver sections from early fibrotic 2W and 4W mice injected with Fe_3_O_4_/Gd@BSA‐pPB probe for 60 min. Scale bar = 1 mm, Magnified image = 200 nm. L,M) Fluorescence intensity analysis and half‐life curves of liver sections at different time points after Fe_3_O_4_/Gd@BSA‐pPB probe injection. The data are shown as the means ± SEM. ^*^ *p* < 0.05, ^**^ *p* < 0.01, ^***^ *p* < 0.001, ^****^ *p* < 0.0001; 2‐tailed Student's *t*‐test (B), 2‐way ANOVA (D, E, M).

Establishment of early nonalcoholic fatty liver fibrosis and validation of ultrasensitive Fe_3_O_4_/Gd@BSA‐pPB probe targeting in pathological situations. Motivated by the results of our cellular experiments, we applied the Fe_3_O_4_/Gd@BSA‐pPB probe in a NASH model of early fibrosis to assess its specificity for targeting the affected regions. We established various stages of early hepatic fibrosis in C57BL/6J mice through a high‐fat diet combined with intraperitoneal injections of 5% CCl_4_ administered twice weekly for 2, 4, and 6 weeks, using 5‐week‐old female mice as subjects.^[^ ^51^ ^]^ The mice fed solely a high‐fat diet served as controls. Serum samples were collected at 7, 9, and 11 weeks for biochemical analysis, while liver tissues were assessed for relevant fibrosis markers (**Figure** **4**A). Our findings revealed minimal differences in serum biochemistry markers among mice with induced early liver fibrosis (Figure 4B; Figures S12 and S13, Supporting Information). In the liver, as fibrosis progresses, liver weight is elevated, HE damage increases, and the liver becomes progressively whiter, probably due to lipid deposition and fibrosis. (Figure 4C–E). The expression levels of α‐SMA and PDGFRβ proteins increased in correlation with the frequency of CCl_4_ administration (Figure 4F,G). The above results suggest that routine blood testing may not be an effective tool for diagnosing early liver fibrosis compared to liver biopsy (phenotyping, HE assay), α‐SMA, and PDGFRβ protein expression assays. We developed an ultrasensitive Fe_3_O_4_/Gd@BSA‐pPB probe that specifically binds to PDGFRβ protein, which exhibits progressively higher expression in liver fibrosis. As fibrosis severity and LX‐2 cell activation increased, the expression of PDGFRβ was elevated, leading to enhanced binding of the ultrasensitive Fe_3_O_4_/Gd@BSA‐pPB probe in the affected areas (Figure 4I). The progression of liver fibrosis was further verified through fluorescence imaging of the Cy5.5‐labeled ultrasensitive Fe_3_O_4_/Gd@BSA‐pPB probe, which selectively targets PDGFRβ upregulated by activated HSCs. We acquired in vivo fluorescence images of mice at various time points, ranging from 5 to 60 min, as shown in Figure 4H. These images confirm the accumulation of the ultrasensitive Fe_3_O_4_/Gd@BSA‐pPB probe at the site of early liver fibrosis. Over time, fluorescence in the focal area of the liver gradually increased, with accelerated fluorescence enrichment coinciding with the intensification of fibrosis (from 7 to 11 weeks). At 60 min, fluorescence intensity was significantly deeper. The fluorescent probe accurately reflected both the location of the fibrotic area and the severity of the disease (Figure 4J). We selected liver samples from 2W, and 4W fibrotic mice 60 min post‐injection for TEM image collection and ICP‐MS testing further supported these findings (Figure 4K; Figure S14, Supporting Information). We conducted immunohistochemical analysis on liver samples from 2W fibrotic mice at various time points (5 to 90 min) following injection. As illustrated in Figure 4L, the Cy5.5 fluorescence signals (red) in the liver regions of the ultrasensitive Fe_3_O_4_/Gd@BSA‐pPB probe were consistently higher than those of the BSA‐pPB group at all time points. Notably, fluorescence intensity assays (Figure 4M) demonstrated that Fe_3_O_4_/Gd@BSA‐pPB exhibited superior targeting ability and a prolonged half‐life in the liver, whereas free BSA‐pPB was rapidly metabolized (showing only one‐third of the fluorescence intensity of Fe_3_O_4_/Gd@BSA‐pPB). Compared with the single binding mode of BSA‐pPB, the abundant binding sites on the surface of Fe_3_O_4_/Gd@BSA‐pPB probe resulted in a stronger affinity for PDGFRβ, which might be responsible for the increased half‐life and sensitivity of the ultrasensitive probe.

### 2.4 Ultrasensitive Fe_3_O_4_/Gd@BSA‐pPB Probe for Diagnostic MR Imaging(7T) of Early Nonalcoholic Fatty Liver Fibrosis

**Figure 5:**
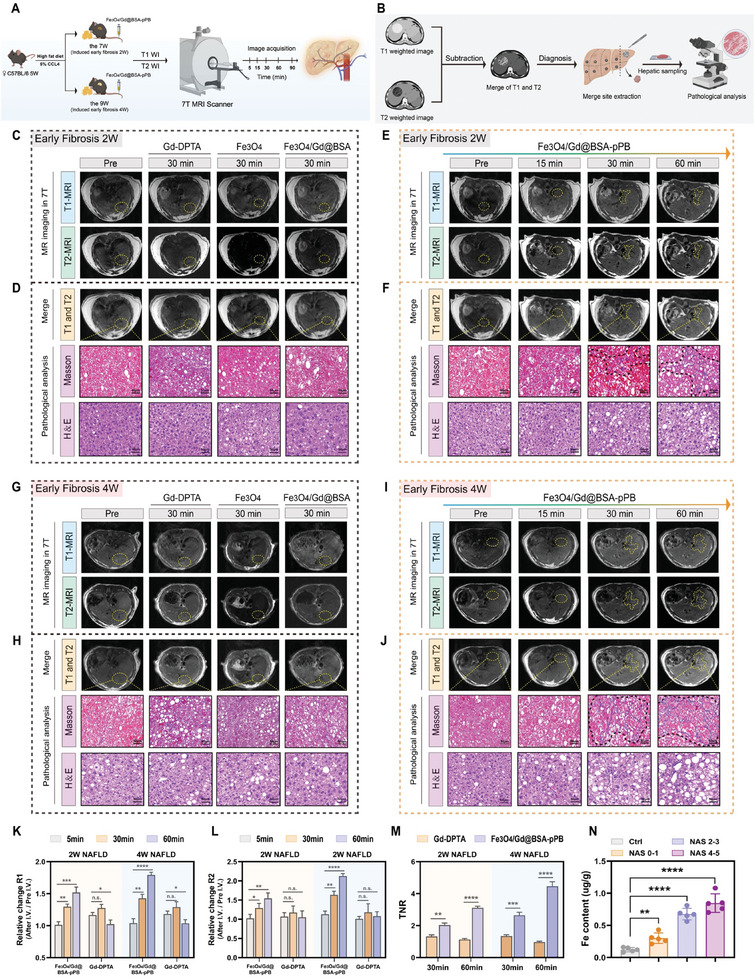
Ultrasensitive Fe_3_O_4_/Gd@BSA‐pPB probe for diagnostic MR imaging(7T) of early nonalcoholic fatty liver fibrosis. A) Schematic illustration for MR imaging‐based diagnosis. After intravenous injection of Gd‐DPTA, ultrasensitive Fe_3_O_4_/Gd@BSA‐pPB probe (10 mg Fe kg^−1^), or Fe_3_O_4_ particles (10 mg Fe kg^−1^), early‐stage nonalcoholic fatty liver fibrosis mice (induced for 2 and 4 weeks, n = 3/group) were subjected to different times of MR image acquisition. B) Illustration of dual‐contrast enhanced imaging procedures, involving the injection of different contrast agents followed by acquiring T_1_ and T_2_ MR images at different times, respectively C,E,G,I). The merge images, Masson's pathology, and HE‐staining slides are shown that D,F) in the early fibrosis 2W and H,J) in the early fibrosis 4W. R_1_ K), R_2_ L), and TNR M) were obtained and calculated for the diagnosed livers of different groups. N) Correlation analysis between Fe content and NAS score of the mice liver. The data are shown as the means ± SEM. ^*^ *p* < 0.05, ^**^ *p* < 0.01, ^***^ *p* < 0.001, ^****^ *p* < 0.0001; 2‐tailed Student's *t*‐test (M), 2‐way ANOVA (K, L, N), Scale bar = 50 µm.

The Fe_3_O_4_/Gd@BSA‐pPB probe demonstrated superior dual T_1_‐ and T_2_‐weighted imaging capabilities, along with acute recognition and tight binding properties to LX‐2 cell activation, making it a promising candidate for the diagnosis of early‐stage nonalcoholic fatty liver fibrosis in vivo. As illustrated in **Figure** **5**A, we employed the previously described method to construct a model of early‐stage NAFLD fibrosis. Due to the combination of a high‐fat diet and mild early‐stage fibrotic disease, the detection of fibrotic changes through serologic testing in mice with NAFLD is nearly impossible (Figure 4B; Figures S12 and S13, Supporting Information). We injected the ultrasensitive Fe_3_O_4_/Gd@BSA‐pPB probe (10 mg Fe kg^−1^) into 7 weeks (early fibrosis 2 weeks‐) and 9 weeks (early fibrosis 4 weeks‐) mice via the tail vein, respectively, and performed simultaneous T_1_ and T_2_ image acquisition at various time points within 60 min using a 7T MRI scanner (Bruker BioSpec 70/30).^[^ ^52^ ^]^ The T_1_ and T_2_ images acquired concurrently were combined for dual‐modality contrast imaging analysis, and the identified Fibrotic sites were subjected to tissue extraction, Masson ʹs trichrome staining, and HE‐staining slides to assess the accuracy and sensitivity of the different detection methods (Figure 5B). In addition, Gd‐DPTA is a commercially available T_1_ contrast agent, while Fe_3_O_4_ particles are a typical T_2_ contrast agent. They represent two different imaging means (T_1_ or T_2_, respectively) for the diagnosis of liver fibrosis.

As shown in Figure 5C,G, commercial Gd‐DTPA was ineffective for NAFLD imaging, as evidenced by only a slight brightening in the T_1_ image at 30 min and minimal change in the T_2_ image (Figure S15, Supporting Information). The short half‐life was inadequate to navigate the complex NAFLD environment, and the insufficient targeting ability contributed to the poor imaging performance of Gd‐DTPA. Following injection, Fe_3_O_4_ particles were taken up and deposited in the liver, resulting in low resolution of T_2_ images throughout the acquisition and negligible changes in T_1_ images. Gd‐DTPA and Fe_3_O_4_ particles were analyzed by MRI dual‐mode contrast imaging to identify the site, and the lack of fibrosis detected in the pathology section supports the above conclusion (Figure 5D,H). Similarly, Fe_3_O_4_/Gd@BSA was not able to effectively distinguish fibrotic regions in early‐stage fibrosis (Figures S16–S18, Supporting Information). After the ultrasensitive Fe_3_O_4_/Gd@ BSA‐pPB probe was injected into mice via the tail vein, T_1_ and T_2_ changes were not obvious at 15 min, during which the nanoprobe circulated in the bloodstream and gradually recognized and accumulated in the fibrotic region of the liver. As time progressed, at 30 min, the T_1_ and T_2_ signals in the liver region intensified, illuminating the image and allowing for a vague visualization of the fibrotic area. At 60 min, the T_1_ and T_2_ images were further enhanced, rendering the fibrotic area clearer (Figure 5E,F). We conducted a dual‐modality contrast imaging analysis based on the acquired T_1_ and T_2_ images, followed by Massonʹs trichrome and HE staining of the identified area, which revealed severe fibrosis (Figure 5I,J). Surprisingly, in early fibrosis 4W mice, the T_1_ and T_2_ images acquired using the ultrasensitive Fe_3_O_4_/Gd@BSA‐pPB probe were clearer (Yellow dotted line marking), and the diagnosed site exhibited greater fibrosis (Black dotted line marking). This finding suggests that the ultrasensitive Fe_3_O_4_/Gd@BSA‐pPB probe is capable of tracing early fibrosis during disease progression. After the obtained images were quantified, as shown in Figure 5K,L, the ultrasensitive Fe_3_O_4_/Gd@BSA‐pPB probe exhibited a 1.5‐fold enhancement in both R_1_ and R_2_ values in early fibrotic livers at 60 min post‐injection in early fibrosis 2W mice. In 4W mice, this enhancement was even more pronounced, with R_1_ values elevated by 1.78‐fold and R_2_ values by 2.12‐fold. In contrast, commercial Gd‐DTPA exhibited minimal changes in R_1_ and R_2_ values. Dual‐modality contrast imaging analysis revealed that the fibrotic site versus normal liver tissue signal ratio (TNR) was 3.0‐fold in 2W mice and 4.46‐fold in 4W mice (Figure 5M). Furthermore, the positive correlation between Fe content and the NAS suggests that the developed Fe_3_O_4_/Gd@BSA‐pPB probe possesses excellent targeting capability for efficiently diagnosing disease progression (Figure 5N).

### 2.5 In Vivo Distribution and Biocompatibility Assessment of the Ultrasensitive Fe_3_O_4_/Gd@BSA‐pPB Probe

**Figure 6:**
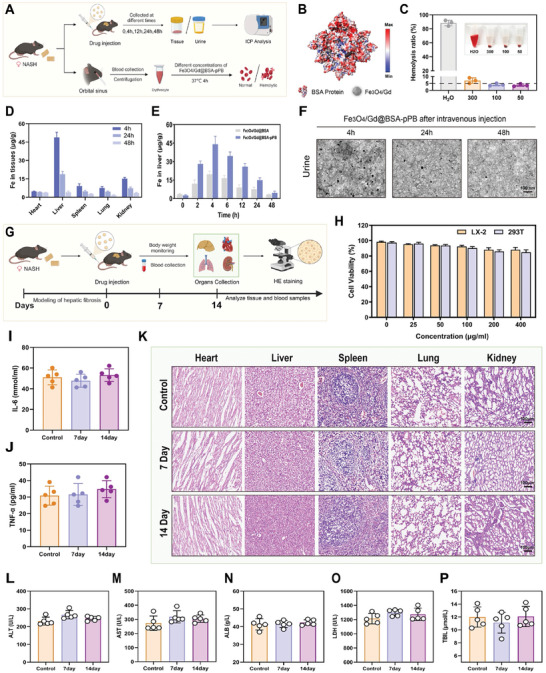
In vivo distribution and biocompatibility assessment of the ultrasensitive Fe_3_O_4_/Gd@BSA‐pPB probe. A) Schematic diagram of tissue distribution assay and hemolysis test (10 mg Fe kg^−1^, n = 5). B) Surface charge distribution of Fe_3_O_4_/Gd@BSA‐pPB. C) Hemolysis caused by different concentrations (300, 100, 50 µg mL^−1^) of ultra‐sensitive Fe_3_O_4_/Gd@ BSA‐pPB probe after incubation with red blood cells. D) Time‐dependent biodistribution analysis of Fe in major organs 4, 24, and 48 h after injection. E) Changes in hepatic Fe content in mice with early fibrotic livers by ultrasensitive Fe_3_O_4_/Gd@BSA‐pPB probe and Fe_3_O_4_/Gd@BSA. F) TEM images of urine after 4,6 and 24 h after injection, Scale bar = 100 nm. G) The schematic diagram for the bio‐compatibility evaluation of ultrasensitive Fe_3_O_4_/Gd@BSA‐pPB probe (10 mg Fe kg^−1^, n = 5). H) The cell viability assays of LX‐2 cells and 293T cells. Inflammatory factor IL‐6 I) and TNF‐α J) changes in serum after injection. K) H&E analysis of the major organs after injection. Scale bar = 100 µm. L–P) Major blood biochemical parameters were measured 7 and 14 days after injection. ALT: alanine transaminase, AST: aspartate transaminase, ALB: albumin, LDH: lactate dehydrogenase, TBIL: total bilirubin. The data are shown as the means ± SEM. The statistical significance of differences was determined by 2‐way ANOVA.

With the advancement of nanotechnology, an increasing number of contrast agents are being utilized in the clinic, among which gadolinium (Gd)–based MR contrast agents have been used to differentiate NASH from fatty liver or hepatic steatosis.^[^ ^53^ ^]^ However, gadolinium‐based contrast agents are associated with significant risks, including nephrotoxicity, which can lead to nephrogenic systemic fibrosis, particularly in individuals with impaired renal function.^[^ ^54^, ^55^ ^]^ The introduction of BSA has resulted in an ultrasensitive Fe_3_O_4_/Gd@BSA‐pPB probe with excellent stability, low immunogenicity, and excellent biocompatibility. As illustrated in **Figure** **6**A, in mice with induced early fibrosis, the Fe_3_O_4_/Gd@BSA‐pPB probe was injected via the tail vein at a dose of 10 mg Fe kg^−1^. Tissues and urine were collected at 0, 4, 12, 24, and 48 h for ICP‐MS measurements of Fe and Gd. Additionally, erythrocytes were collected via orbital blood sampling and incubated with varying concentrations of the Fe_3_O_4_/Gd@BSA‐pPB probe at 37 °C for 4 h in vitro to assess erythrocyte hemolysis. The stability experiments demonstrated that the Fe_3_O_4_/Gd@BSA‐pPB probe remained stable for a long time even at high concentrations in vitro (Figures S19 and S20, Supporting Information). This stability can be attributed to the abundant hydrophilic residues on the BSA proteins that are randomly exposed on the outer surface, facilitating the nanoprobe's interactions with water and enhancing the stability of the aqueous solution (Figure 6B).^[^ ^56^ ^]^ Hemolysis experiments were conducted to assess the effect of Fe_3_O_4_/Gd@BSA‐pPB probes with varying iron concentrations on erythrocytes (Figure 6C). The results indicated that the ultrasensitive Fe_3_O_4_/Gd@BSA‐pPB probes exhibited high compatibility with hemocytes, even at concentrations as high as 300 µg mL^−1^. Over time, the ultrasensitive Fe_3_O_4_/Gd@BSA‐pPB probe primarily accumulated in the liver due to the targeting ability of pPB, and it was nearly completely metabolized by the liver within 48 h. Other tissues also cleared the Fe_3_O_4_/Gd@BSA‐pPB probe after this time frame (Figure 6D,E; Figure S21 and S22, Supporting Information). The Fe concentration in the urine of mice increased and subsequently decreased after injection, with nearly all of it cleared after 48 h in vivo (Figure 6F). To further validate the compatibility of the Fe_3_O_4_/Gd@BSA‐pPB probe in vivo, we similarly injected Fe_3_O_4_/Gd@BSA‐pPB probe into the tail vein of early fibrotic mice, and serum was collected for biochemical analyses and tissues of each site were used for HE sections on days 0, 7, 14, respectively (Figure 6G). A series of Fe_3_O_4_/Gd@BSA‐pPB probes were safe for both LX‐2 and 293T (Figure 6H). The post‐injection Fe_3_O_4_/Gd@BSA‐pPB probe did not induce an inflammatory response in mice (Figure 6I,J), and no damage was observed in the HE results of the major organs (heart, liver, spleen, lungs, and kidneys) (Figure 6K), and there were no differences in the changes of blood biochemical parameters compared with the pre‐injection (Figure 6L–P; Figure S23, Supporting Information). The above findings indicate that the Fe_3_O_4_/Gd@BSA‐pPB probe has excellent stability and compatibility in vivo, which is attributed to the formation of biomineralization through BSA proteins.

## 3 Conclusion

In this study, we developed an ultrasmall MRI probe, Fe_3_O_4_/Gd@BSA‐pPB (≈5 nm), aimed at enhancing the diagnostic sensitivity of MRI techniques for early‐stage NAFLD fibrosis. This probe is based on a protein‐cage structure and exhibits dual activation signals for T_1_ and T_2_ imaging. This approach integrates protein‐mediated biomimetic synthesis with the targeted identification of specific surface markers linked to early liver fibrosis, facilitating the tracking of disease progression. In this structure, Gd biomineralized with Fe_3_O_4_, is successfully encapsulated into the cage‐like structure formed by BSA, which takes advantage of the structural stability of BSA as well as the presence of a large number of hydrophilic residues, greatly facilitating the interaction of the nanoprobe with water and prolonging its half‐life in the blood circulation. The surface‐modified specific small peptides can help the Fe_3_O_4_/Gd@BSA‐pPB probe to accurately and rapidly enrich the focal region of the liver to achieve contrast imaging analysis in both T_1_ and T_2_ modalities (TNR up to 4.46‐fold). Blockade of receptor PDGFRβ by nanoprobes may inhibit the exacerbation of liver fibrosis. In vitro and in vivo biochemical analyses and toxicity tests, in particular renal clearance tests, confirmed the high biocompatibility. In response to the lack of difference in serologic testing in early liver fibrosis, the non‐invasive approach of the ultrasensitive Fe_3_O_4_/Gd@BSA‐pPB probe on a 7T MRI instrument not only improves the clarity and contrast of the fibrous area but also the entire assay can be completed in less than 60 min, which greatly facilitates the clinical application. Overall, this study provides a design idea for a contrast agent that can improve the sensitivity of early liver fibrosis detection in NAFLD, which is clinically important in the early diagnosis, prognosis, and recurrence monitoring of the disease.

## 4 Experimental Section

### Materials

FeCl_3_·6H_2_O, FeSO_4_·7H_2_O, CdCl_2_, BSA protein, and NaOH were obtained from Sigma–Aldrich. 1‐(3‐(Dimethylamino) propyl)‐3‐ethylcarbodiimide hydroch‐loride (EDC) and N‐hydroxysuccinimide (NHS) were sourced from Sangon Biotech Co., Ltd. (China). The CCK‐8 (Cell Counting Kit‐8) and the live/dead cell staining kit were acquired from Bestbio, Shanghai. α‐SMA, PDGFRβ antibody, and TGF‐β were procured from Abcam. All ELISA kits were obtained from Thermo Fisher Scientific. All reagents used in the experiments were of at least analytical grade and did not require further purification.

### Synthesis of Fe_3_O_4_/Gd@BSA Nanoparticles

In this experiment, we synthesized BSA protein cage‐encapsulated Fe_3_O_4_/Gd using a chemical co‐precipitation method. Briefly, a BSA solution was mixed with an iron/gadolinium solution (Fe^3^⁺/Fe^2^⁺ = 2:1, GdCl_2_: 35 mm) under nitrogen protection in a 37 °C water bath. NaOH solution at a concentration of 100 mm was then added to the mixture at a constant rate of 12 mL h^−1^. The reaction was allowed to proceed for 30 min. The resulting products were centrifuged at 12 000 rpm for 15 min, filtered through a 0.22 µm membrane to remove bacteria, and stored in sterile tubes at room temperature (RT).^[^ ^57^, ^58^ ^]^

### Synthesis of Fe_3_O_4_/Gd@BSA‐pPB Probes

A total of 100 µL of prepared Fe_3_O_4_/Gd@BSA nanoparticles was added to a 900 µL solution containing N‐hydroxysu‐ccinimide (NHS) and 1‐ethyl‐3‐[3‐dimethylaminopropyl] carbodiimide hydro‐chloride (EDC) (10 mg mL^−1^, dissolved in MES, 0.5 M, pH 5.0). The mixture was stirred for 10 min and then concentrated using a 100 KDa concentrator tube to remove residual NHS, EDC, and reaction by‐products. The pPB cyclic peptide was subsequently added at a molar ratio of 1:10 and reacted for 2 h at room temperature on a rotary stirrer. The mixture was then washed three times with PBS to eliminate unreacted pPB. The prepared Fe_3_O_4_/Gd@BSA‐pPB probe was stored at 4 °C for future use.

### MRI Performance

T_1_‐weighted imaging (T_1_WI), T_2_‐weighted imaging (T_2_WI), T_1_ maps, and T_2_ maps were acquired using a 3.0 T MRI scanner (Philips Medical Systems, the Netherlands) across various cell types and concentrations. The acquisition parameters were as follows: T_1_‐weighted MR images were obtained using a turbo spin echo (TSE) sequence with parameters of TR/TE = 150, 300, 600, 1000, 2000, 4000, 8000/11 ms; slice thickness of 3 mm; flip angle of 90°; average number of signals of 2; and field of view (FOV) of 120 mm × 120 mm with a matrix size of 240 × 240. T_2_‐weighted MR images were obtained using a multi‐spin echo (MSE) sequence with similar parameters, except for TR/TE values of 5000/10, 20, 30, 40, 50, 60, 70, 80, 90, and 100 ms. To evaluate cell specificity in vitro, we selected different cell lines to represent varying degrees of fibrosis. Renal epithelial cells (293T), hepatocellular carcinoma cells (HepG.2), and activated stellate cells (LX‐2) were seeded in 6 cm cell culture dishes at a density of 1 × 10⁶ cells per well and cultured for 12 h. Fe_3_O_4_/Gd@BSA‐pPB (0.1 mg/mL) was then added to each well and incubated for 4 h at 37 °C. And then, the MRI performances were measured according to the method mentioned above. In vivo imaging of mice was performed on a 7T MRI scanner (Bruker BioSpec 70/30) with the following imaging parameter settings: T_2_ WI:TR = 1187.39 ms, TE = 24.0, FOV = 30 mm × 20 mm, matrix = 200 × 200; T_1_ WI:TR = 1500 ms, TE = 6.5 ms, FOV = 30 mm × 20 mm, matrix = 300 × 192; T_1_ WI:TR = 1500 ms, TE = 6.5 ms, FOV = 30 mm × 20 mm, FOV = 30 mm × 20 mm.

### Surface Plasmon Resonance

The experiments were conducted at room temperature using a Biacore T200 (GE Healthcare) equipped with a CM5 sensor chip (GE Healthcare, Cat# BR‐1005‐30). Target proteins were coupled to the CM5 sensor chip through EDC/NHS chemistry. In kinetic assays, PDGFRβ proteins were immobilized on the sensor chip following the injection of 2‐fold serial dilutions of either Fe_3_O_4_/Gd@BSA‐pPB Probe or BSA‐pPB. After each injection, the chip surface was regenerated with 350 mM EDTA and 50 mm NaOH before the immobilization of the ligand for the subsequent injection. Consistency was maintained by performing all experiments for each ligand on the same channel. An empty channel was used as a reference for injecting the running buffer, from which the Kon, Koff, and KD values were calculated. The experiments were repeated with freshly prepared reagents, and the data were analyzed using Software Control (version 2.0.1) and BIA Evaluation Software (version 3.0).

### In Vivo Biocompatibility

The biocompatibility of Fe_3_O_4_/Gd@BSA was assessed through weight monitoring, blood biochemical analysis, and histopathological examination of major organs. In the experimental group, mice were injected intravenously with Fe_3_O_4_/Gd@BSA at a dosage of 10 mg Fe kg^−1^, while the control group received an equivalent volume of PBS (n = 5). The body weight of the mice was recorded post‐injection, and serum samples were collected at various time points (0, 7, and 14 days) for biochemical analysis. The biochemical tests included liver function indicators such as alanine aminotransferase (ALT), serum albumin (ALB), aspartate aminotransferase (AST), and total protein (TP), along with kidney function indices including serum creatinine (CREA), blood urea nitrogen (BUN), and uric acid (UA). Furthermore, major organs (brain, heart, liver, spleen, lungs, and kidneys) were evaluated across different groups (control, 7 and 14 days). The corresponding tissues were excised, fixed in a 4% formaldehyde solution, and subjected to HE staining.

### Statistical Analysis

All statistical analyses and preprocessing were performed using SPSS 16.0 and/or GraphPad Prism software. Experimental data were presented as the mean ± standard deviation (SD). In vivo experiments on mice, n = 5 was the minimum number of animals used. For cell studies, the data were representative of at least three independent experiments. Student's t‐test was used to compare whether there was a difference between two groups, one‐way ANOVA test was used to evaluate the difference among three or more groups. *p* < 0.05 was defined as statistically significant. ^*^, ^**^, ^***^ and ^****^ represent *p* < 0.05, *p* < 0.01, *p* < 0.001, *p* < 0.0001, respectively.

## Conflict of Interest

The authors declare no conflict of interest.

## Supporting information



Supporting Information

## Data Availability

The data that support the findings of this study are available in the supplementary material of this article.

## References

[1] S. Pouwels , N. Sakran , Y. Graham , A. Leal , T. Pintar , W. Yang , R. Kassir , R. Singhal , K. Mahawar , D. Ramnarain , BMC Endocr. Disord. 2022, 22, 63. 35287643 10.1186/s12902-022-00980-1 PMC8919523

[2] Z. M. Younossi , G. C. Wong , Q. M. Anstee , L. Henry , Clin. Gastroenterol. Hepatol. 2023, 21, 1978. 37121527 10.1016/j.cgh.2023.04.015

[3] K. S. Kim , S. M. Hong , K. Y. D. Han , C. Y. Park , Bmj 2024, 384, e076388. 38350680 10.1136/bmj-2023-076388 PMC10862140

[4] C. L. Hsu , R. Loomba , Nat. Metab. 2024, 6, 600. 38383845 10.1038/s42255-024-00985-1 PMC11262457

[5] S. H. Ibrahim , R. Kohli , G. J. Gores , J. Pediatr. Gastroenterol. Nutr.n 2011, 53, 131. 10.1097/MPG.0b013e31822578db PMC3145329 21629127

[6] L. Wiering , F. Tacke , J. Endocrinol. 2023, 256, 20. 10.1530/JOE-22-0194 36259984

[7] A. J. Sanyal , J. Foucquier , Z. M. Younossi , S. A. Harrison , P. N. Newsome , W. K. Chan , Y. Yilmaz , V. De Ledinghen , C. Costentin , M. H. Zheng , V. W. S. Wong , M. Elkhashab , R. S. Huss , R. P. Myers , M. Roux , A. Labourdette , M. Destro , C. Fournier‐Poizat , V. Miette , L. Sandrin , J. Boursier , J. Hepatol. 2023, 78, 247. 36375686 10.1016/j.jhep.2022.10.034 PMC10170177

[8] V. Ajmera , S. Cepin , K. Tesfai , H. Hofflich , K. Cadman , S. Lopez , E. Madamba , R. Bettencourt , L. Richards , C. Behling , C. B. Sirlin , R. Loomba , J. Hepatol. 2023, 78, 471. 36410554 10.1016/j.jhep.2022.11.010 PMC9975077

[9] M. A. Karim , A. G. Singal , H. C. Kum , Y. T. Lee , S. Park , N. E. Rich , M. Noureddin , J. D. Yang , Clin. Gastroenterol. Hepatol. 2023, 21, 670. 35307595 10.1016/j.cgh.2022.03.010 PMC9481743

[10] F. Nassir , Biomolecules 2022, 12, 824. 35740949 10.3390/biom12060824 PMC9221336

[11] T. Ito , V. H. Nguyen , T. Tanaka , H. Park , M. L. Yeh , M. Kawanaka , T. Arai , M. Atsukawa , E. L. Yoon , P. C. Tsai , H. Toyoda , J. F. Huang , L. Henry , D. W. Jun , M. L. Yu , M. Ishigami , M. H. Nguyen , R. C. Cheung , Clin. Gastroenterol. Hepatol. 2023, 21, 1013. 35654298 10.1016/j.cgh.2022.05.015

[12] S. Condon , H. R. Hu , M. Y. Kong , M. C. Cave , C. J. McClain , Am. J. Med. Sci. 2024, 367, 310. 38307172 10.1016/j.amjms.2024.01.022 PMC11299156

[13] D. Q. Huang , L. A. Wilson , C. Behling , D. E. Kleiner , K. Kowdley , S. Dasarathy , M. Amangurbanova , N. A. Terrault , A. M. Diehl , N. Chalasani , B. A. Neuschwander‐Tetri , A. J. Sanyal , J. Tonascia , R. Loomba , N. C. R. Network , Gastroenterology 2023, 165, 463.37127100

[14] P. S. Dulai , C. B. Sirlin , R. Loomba , J. Hepatol. 2016, 65, 1006. 27312947 10.1016/j.jhep.2016.06.005 PMC5124376

[15] M. Noureddin , E. Truong , J. A. Gornbein , R. Saouaf , M. Guindi , T. Todo , N. Noureddin , J. D. Yang , S. A. Harrison , N. Alkhouri , J. Hepatol. 2022, 76, 781. 34798176 10.1016/j.jhep.2021.11.012

[16] E. A. Selvaraj , F. E. Mózes , A. N. A. Jayaswal , M. H. Zafarmand , Y. Vali , J. A. Lee , C. K. Levick , L. A. J. Young , N. Palaniyappan , C. H. Liu , G. P. Aithal , M. Romero‐Gómez , M. J. Brosnan , T. A. Tuthill , Q. M. Anstee , S. Neubauer , S. A. Harrison , P. M. Bossuyt , M. Pavlides , J. Hepatol. 2021, 75, 770. 33991635 10.1016/j.jhep.2021.04.044

[17] Y. Ding , S. X. Rao , T. Meng , C. Z. Chen , R. C. Li , M. S. Zeng , Eur. Radiol. 2014, 24, 959. 24463697 10.1007/s00330-014-3096-y

[18] S. Yan , K. Hu , M. Zhang , J. Y. Sheng , X. Q. Xu , S. J. Tang , Y. Li , S. Yang , G. X. Si , Y. Mao , Y. Zhang , F. M. Zhang , N. Gu , Bioact. Mater. 2023, 19, 418. 35574059 10.1016/j.bioactmat.2022.04.024 PMC9079175

[19] N. Guidolin , F. Travagin , G. B. Giovenzana , A. Vágner , S. Lotti , F. Chianale , E. Brücher , F. Maisano , M. A. Kirchin , F. Tedoldi , A. Giorgini , S. C. Serra , Z. Baranyai , Dalton Trans. 2020, 49, 14863. 33073806 10.1039/d0dt03314f

[20] R. X. Wei , K. Liu , K. Zhang , Y. F. Fan , H. Y. Lin , J. H. Gao , ACS Appl. Mater. Interfaces 2022, 14, 3784. 35019261 10.1021/acsami.1c20617

[21] H. D. Chen , X. D. Li , F. Y. Liu , H. M. Zhang , Z. X. Wang , Mol. Pharm. 2017, 14, 3134. 28727430 10.1021/acs.molpharmaceut.7b00361

[22] J. Sobhanan , A. Anas , V. Biju , Chem. Rec. 2023, 23, e202200253. 36789795 10.1002/tcr.202200253

[23] B. C. Jiang , F. Liu , H. D. Fu , J. H. Mao , Ren. Fail. 2023, 45, 2171887. 36723057 10.1080/0886022X.2023.2171887 PMC9897785

[24] D. Dhar , J. Baglieri , T. Kisseleva , D. A. Brenner , Exp. Biol. Med. 2020, 245, 96. 10.1177/1535370219898141 PMC7016420 31924111

[25] A. Elzoheiry , E. Ayad , N. Omar , K. Elbakry , A. Hyder , Sci. Rep. 2022, 12, 18403. 36319750 10.1038/s41598-022-23276-9 PMC9626641

[26] X. Y. Xue , X. T. Zhao , J. Wang , C. Wang , C. Ma , Y. F. Zhang , Y. X. Li , C. Peng , Phytomedicine 2023, 108, 154517. 36332390 10.1016/j.phymed.2022.154517

[27] L. Wu , X. Q. Huang , N. Li , C. Xie , S. X. Rao , S. Y. Chen , F. Li , J. Gastroenterol. Hepatol. 2021, 36, 3448. 34278598 10.1111/jgh.15628

[28] J. Lambrecht , S. Verhulst , I. Mannaerts , J. P. Sowa , J. Best , A. Canbay , H. Reynaert , L. A. van Grunsven , Ebiomedicine 2019, 43, 501. 31036530 10.1016/j.ebiom.2019.04.036 PMC6558023

[29] J. J. Liu , X. D. Yu , H. J. Ting , X. Y. Wang , S. D. Xu , Y. B. Wang , S. T. Zhang , J. W. Wang , B. Liu , ACS Nano 2023, 17, 3324. 36773320 10.1021/acsnano.2c06233

[30] Q. Wan , H. Peng , J. X. Lyu , F. Liu , C. A. L. Cheng , Y. Z. Qiao , J. Deng , H. R. Zheng , Y. Wang , C. Zou , X. Liu , J. Magn. Reson. Imaging 2022, 56, 1429. 35212074 10.1002/jmri.28131

[31] Z. W. Chen , H. M. Xiao , X. J. Ye , K. Liu , R. S. Rios , K. I. Zheng , Y. Jin , G. Targher , C. D. Byrne , J. P. Shi , Z. H. Yan , X. L. Chi , M. H. Zheng , Hepatobiliary Surg. Nutr. 2022, 11, 212. 35464279 10.21037/hbsn-21-23 PMC9023815

[32] H. W. Lu , A. Chen , X. D. Zhang , Z. X. Wei , R. Cao , Y. Zhu , J. X. Lu , Z. L. Wang , L. L. Tian , Nat. Commun. 2022, 13, 7948. 36572677 10.1038/s41467-022-35655-x PMC9792454

[33] S. Z. Zhao , X. J. Yu , Y. N. Qian , W. Chen , J. L. Shen , Theranostics 2020, 10, 6278. 32483453 10.7150/thno.42564 PMC7255022

[34] X. X. Li , W. Y. Li , M. N. Wang , Z. H. Liao , J. Controlled Release 2021, 335, 437. 10.1016/j.jconrel.2021.05.042 34081996

[35] S. Shrestha , B. Wang , P. Dutta , Adv. Colloid Interface Sci. 2020, 279, 102162. 32334131 10.1016/j.cis.2020.102162

[36] M. Hegde , N. Naliyadhara , J. Unnikrishnan , M. S. Alqahtani , M. Abbas , S. Girisa , G. Sethi , A. B. Kunnumakkara , Cancer Lett. 2023, 556, 216066. 36649823 10.1016/j.canlet.2023.216066

[37] H. H. Hwang , D. Y. Lee , in Bioinspired Biomaterials, (Eds: H.J. Chun , R.L. Reis , A. Motta , G. Khang ), Springer, Berlin, Germany 2020, pp. 203–221.

[38] K. Ma , S. Xu , T. X. Tao , J. C. Qian , Q. Q. Cui , S. U. Rehman , X. G. Zhu , R. G. Chen , H. X. Zhao , C. H. Wang , Z. P. Qi , H. Dai , X. Zhang , C. Xie , Y. Lu , H. Z. Wang , J. F. Wang , Proc. Natl. Acad. Sci. USA 2022, 119, e2211228119. 36322742 10.1073/pnas.2211228119 PMC9659412

[39] X. L. Zhao , S. Xu , Y. Jiang , C. H. Wang , S. U. Rehman , S. H. Ji , J. R. Wang , T. X. Tao , H. T. Xu , R. G. Chen , Y. Y. Cai , Y. Y. Jiang , H. Z. Wang , K. Ma , J. F. Wang , Chem. Eng. J. 2023, 454, 140440.

[40] S. Xu , J. R. Wang , Y. Wei , H. X. Zhao , T. X. Tao , H. Wang , Z. Wang , J. Du , H. Z. Wang , J. C. Qian , K. Ma , J. F. Wang , ACS Appl. Mater. Interfaces 2020, 12, 56701. 33296181 10.1021/acsami.0c13825

[41] K. Ma , H. X. Zhao , X. W. Zheng , H. B. Sun , L. Hu , L. Zhu , Y. Shen , T. Luo , H. Dai , J. F. Wang , J. Mater. Chem. B 2017, 5, 2888. 32263982 10.1039/c7tb00570a

[42] J. Yang , J. Feng , S. G. Yang , Y. K. Xu , Z. Y. Shen , Small 2023, 19, 2302856.

[43] B. W. Zhang , T. R. Tian , D. X. Xiao , S. Y. Gao , X. X. Cai , Y. F. Lin , Adv. Funct. Mater. 2022, 32, 2109728.

[44] H. D. Li , H. Kim , C. Zhang , S. Zeng , Q. X. Chen , L. Y. Jia , J. Y. Wang , X. J. Peng , J. Yoon , Coord. Chem. Rev. 2022, 473, 214818.

[45] L. Hammerich , F. Tacke , Nat. Rev. Gastroenterol. Hepatol. 2023, 20, 633. 37400694 10.1038/s41575-023-00807-x

[46] T. X. Tao , Z. H. Li , S. Xu , S. U. Rehman , R. G. Chen , H. T. Xu , H. N. Xia , J. Zhang , H. X. Zhao , J. F. Wang , K. Ma , Anal. Chem. 2023, 95, 11542. 37485962 10.1021/acs.analchem.3c02257

[47] M. Seenuvasan , K. S. Kumar , C. G. Malar , S. Preethi , M. A. Kumar , N. Balaji , Appl. Biochem. Biotechnol. 2014, 172, 2706. 24425303 10.1007/s12010-014-0725-5

[48] D. Tocco , D. Chelazzi , R. Mastrangelo , A. Casini , A. Salis , E. Fratini , P. Baglioni , J. Colloid Interface Sci. 2023, 641, 685. 36965340 10.1016/j.jcis.2023.03.107

[49] C. J. Chen , J. J. Chen , Y. Wang , L. Fang , C. L. Guo , T. T. Sang , H. Peng , Q. Zhao , S. J. Chen , X. J. Lin , X. Y. Wang , Phytomedicine 2023, 110, 154626. 36603342 10.1016/j.phymed.2022.154626

[50] H. Gong , Z. Q. Fan , D. Yi , J. Y. Chen , Z. J. Li , R. Guo , C. J. Wang , W. J. Fang , S. K. Liu , J. Mol. Histol. 2020, 51, 573. 32860079 10.1007/s10735-020-09906-4

[51] G. D. Zhang , X. L. Wang , T. Y. Chung , W. W. Ye , L. Hodge , L. K. Zhang , K. Chng , Y. F. Xiao , Y. J. Wang , Bmc Gastroenterol. 2020, 20, 1. 10.1186/s12876-020-01467-w PMC7560288 33059584

[52] D. Yoon , S. Biswal , B. Rutt , A. Lutz , B. Hargreaves , Muscle Nerve. 2018, 57, 494. 29211916 10.1002/mus.26035 PMC5840304

[53] B. Pulli , G. Wojtkiewicz , Y. Iwamoto , M. Ali , M. W. Zeller , L. Bure , C. H. Wang , Y. Choi , R. Masia , A. R. Guimaraes , K. E. Corey , J. W. Chen , Radiology 2017, 284, 390. 28358240 10.1148/radiol.2017160588 PMC5548451

[54] J. Davies , P. Siebenhandl‐Wolff , F. Tranquart , P. Jones , P. Evans , Arch. Toxicol. 2022, 96, 403. 34997254 10.1007/s00204-021-03189-8 PMC8837552

[55] L. Blomqvist , G. F. Nordberg , V. M. Nurchi , J. O. Aaseth , Biomolecules 2022, 12, 742. 35740867 10.3390/biom12060742 PMC9221011

[56] P. P. Jing , Y. X. Li , Y. H. Su , W. L. Liang , Y. X. Leng , Spectrochim. Acta A‐Mol. Biomol. Spectrosc. 2022, 267, 120604. 34802930 10.1016/j.saa.2021.120604

[57] Z. Deng , M. Xi , C. Zhang , X. R. Wu , Q. G. Li , C. J. Wang , H. P. Fang , G. T. Sun , Y. F. Zhang , G. B. Yang , Z. Liu , ACS Nano 2023, 17, 4495. 36848115 10.1021/acsnano.2c10352

[58] Q. Li , Y. F. Wang , G. Zhang , R. X. Su , W. Qi , Chem. Soc. Rev. 2023, 52, 1549. 36602188 10.1039/d2cs00725h

