# Supplementary material for: Efficient and Specific PDGFRβ‐Targeting Dual‐Mode T1‐T2 MRI Nanoprobe for Early Diagnosis of Non‐Alcoholic Fatty Liver
Source: Adv Sci (Weinh). 2025 Mar 7;12(23):2413788. doi: 10.1002/advs.202413788 (PMC12199383; doi:10.1002/advs.202413788)
Supplement: Supplementary file 1 — Supporting Information [file ADVS-12-2413788-s001.pdf]

ADVANCED SCIENCE
Open Access

Supporting Information

for *Adv. Sci.*, DOI 10.1002/advs.202413788

Efficient and Specific PDGFRβ-Targeting Dual-Mode T_1-T_2 MRI Nanoprobe for Early Diagnosis of Non-Alcoholic Fatty Liver

Zehua Li, Tongwei Zhang, Tongxiang Tao, Yaxuan Liu, Haining Xia, Sajid ur Rehman, Zeyong Guo, Jing Zhang, Ruiguo Chen, Zhan Zhang, Junfeng Wang* and Kun Ma**

Supporting Information

Efficient and Specific PDGFRβ-Targeting Dual-Mode T_1-T_2 MRI Nanoprobe for Early Diagnosis of Non-Alcoholic Fatty Liver

Zehua Li,[‡1,2] Tongwei Zhang,[‡3] Tongxiang Tao,[5] Yaxuan Liu,[3] Haining Xia,[1,2] Sajid ur Rehman,[1] Zeyong Guo,[1] Jing Zhang,[1,2] Ruiguo Chen,[1] Zhan Zhang*[4], Junfeng Wang,*[1,2] Kun Ma*[1]

1. High Magnetic Field Laboratory, Key Laboratory of High Magnetic Field and Ion Beam Physical Biology, Hefei Institutes of Physical Science, Chinese Academy of Sciences, Hefei 230031, Anhui, P. R. China.

2. University of Science and Technology of China, Hefei 230036, Anhui, P. R. China.

3. Key Laboratory of Earth and Planetary Physics, Institute of Geology and Geophysics, Chinese Academy of Sciences, Beijing, 100029, China.

4. Institute of Energy, Hefei Comprehensive National Science Center, Anhui Energy Laboratory, China.

5. College of Pharmacy, Anhui University of Chinese Medicine, 350 Long zi hu Road, Hefei, 230012, China.

[‡]These authors contributed equally to this work.

Experiment Section

Cell Culture and CCK-8 Assay

The 293T and LX-2 cells were cultured in DMEM supplemented with 10% FBS to reach a stable growth phase.[1, 2] The cells were digested with 2% trypsin and suspended to prepare cell suspensions. After counting, the cells were evenly seeded into a 96-well plate at a density of 1000 cells per well (100 μL per well). The plate was incubated at 37°C with 5% CO_2 for 6-8 hours to allow for cell adhesion. Once the cells were fully adhered, the culture medium was aspirated, and varying concentrations of the dual-mode probe Fe_3O_4/Gd@BSA-pPB (400, 200, 100, 50, 25 μg/mL) were added. After 24 hours of co-culture, the culture supernatant was carefully removed, and fresh medium containing 10% CCK-8 solution was added to each well (100 μL). After incubation for 4 hours, absorbance at 450 nm was measured using a microplate reader.[3] Three replicate wells were used for each concentration to ensure reliable experimental results.

Histological staining

The freshly harvested liver was immediately fixed in 10% (v/v) paraformaldehyde. The tissue samples were embedded in paraffin, cut into 4 μm thickness, and stained with hematoxylin and eosin (H&E) as well as Masson's trichrome. Liver samples were frozen in liquid nitrogen and sliced using a cryostat at a thickness of 8 μm. The liver samples were evaluated based on the non-alcoholic fatty liver disease activity score (NAS).[4]

Time-Dependent Biodistribution

Fe_3O_4/Gd@BSA-pPB Probe physiological saline solutions were administered to mice via the tail vein at a dosage of 10 mg Fe/kg. The control group received physiological saline solution alone. Mice were sacrificed at 24h and 48h post-injection, and various organs-including the heart, liver, spleen, lungs, and kidneys-were collected. Following the measurement of body and organ weights, three small tissue samples from different regions of each organ were placed into a 2 mL centrifuge tube to determine the wet weight of each sample. The iron content in each organ was quantified using ICP-MS after acid digestion. To investigate the renal clearance of Fe_3O_4/Gd@BSA-pPB Probe, urine samples from rats were collected at multiple time points (pre-injection, 2, 4, 6, 12, 24, and 48h post-injection) after intravenous administration at a dosage of 10mg Fe/kg. The iron levels in each sample were measured by ICP-MS (Thermo, USA), and nanoparticles in the urine were visualized using TEM (JEM 2100).

The SQUID test

5 mL of the Fe_3O_4/Gd@BSA nanoparticle aqueous solution was centrifuged at high speed to collect the supernatant, which was then freeze-dried for 24 hours. A suitable amount of the freeze-dried sample powder was placed into a capsule carrier, secured with a measuring rod, and inserted into the superconducting quantum interferometer for measurement. The *M-H* curve was determined using the setup program, with testing conducted in segments at a temperature of 300 K and a magnetic field range from 30,000 to 30,000. Upon completion of the sample test, the capsule container was recycled, and the sample powder was extracted to determine the iron concentration via ICP. This procedure facilitated the acquisition of the target sample hysteresis curve.[5]

Supplementary Results

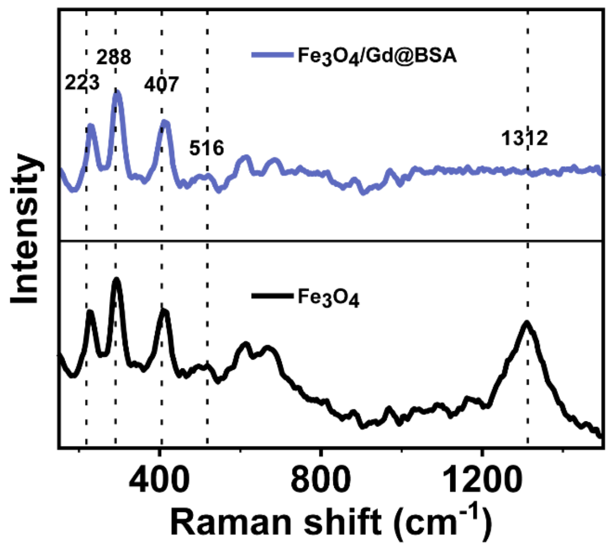

Figure S1: Raman spectra of Fe_3O_4/Gd@BSA and Fe_3O_4 particles.

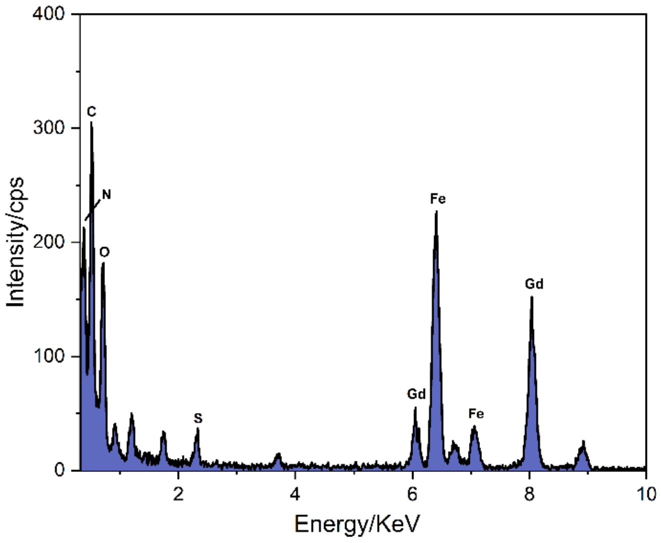

Figure S2: EDS spectra of Fe_3O_4/Gd@BSA.

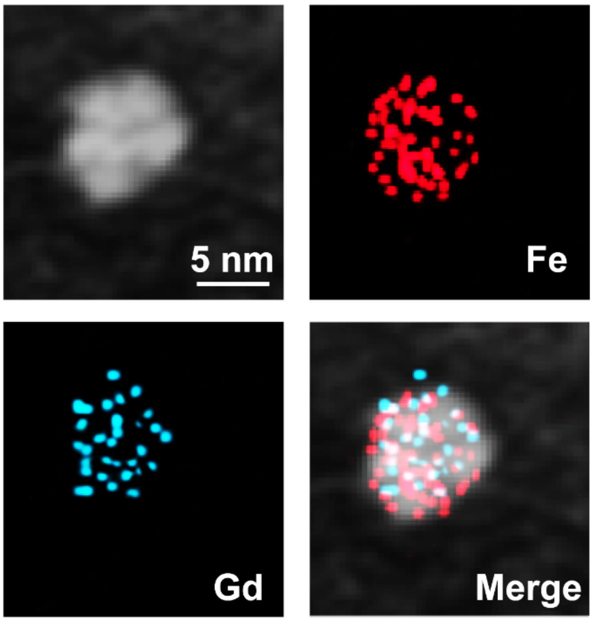

Figure S3: EDX element mapping image of Fe_3O_4/Gd@BSA for Fe, Gd.

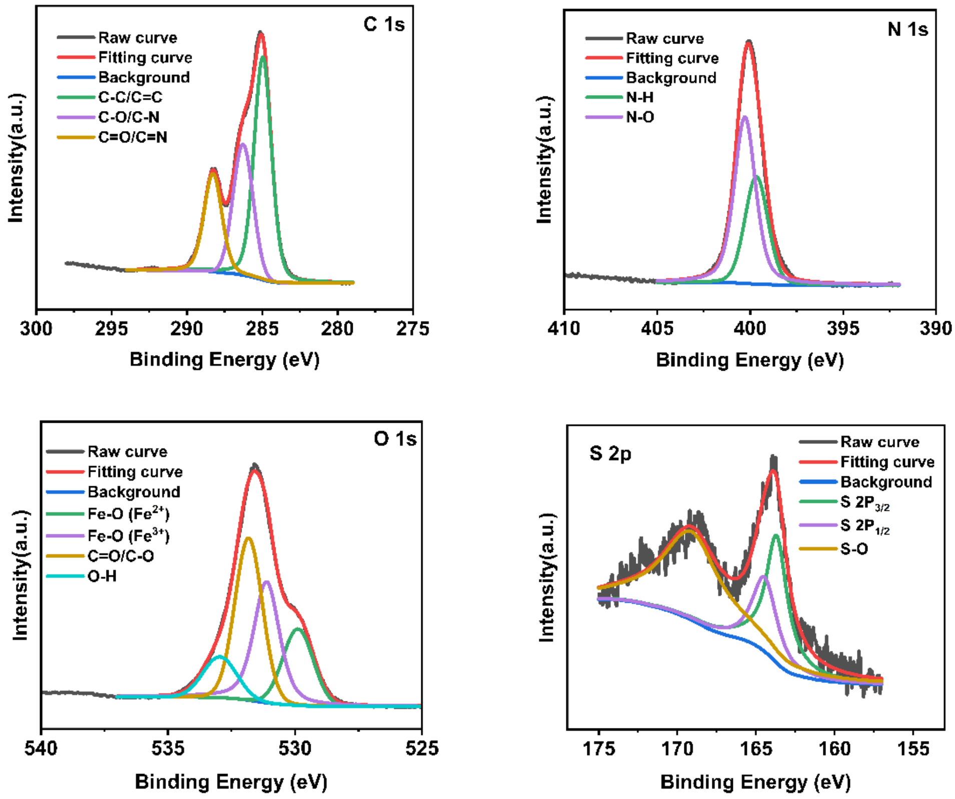

Figure S4: High-resolution XPS spectrum of C 1s, N 1s, O 1s, S 2p.

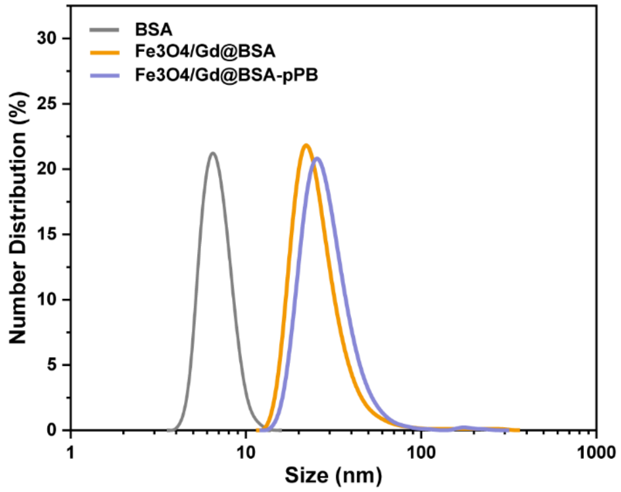

Figure S5: Hydrodynamic diameter of Fe_3O_4/Gd@BSA-pPB probe, Fe_3O_4/Gd@BSA and BSA protein.

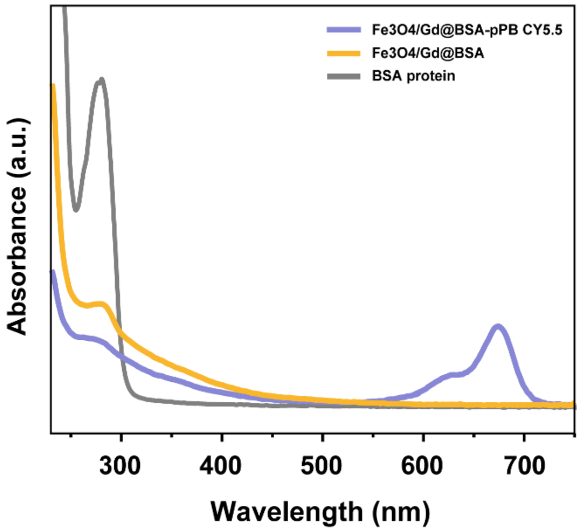

Figure S6: The absorbance 673nm of Fe_3O_4/Gd@BSA-pPB CY5.5 probe, Fe_3O_4 particles and BSA protein.

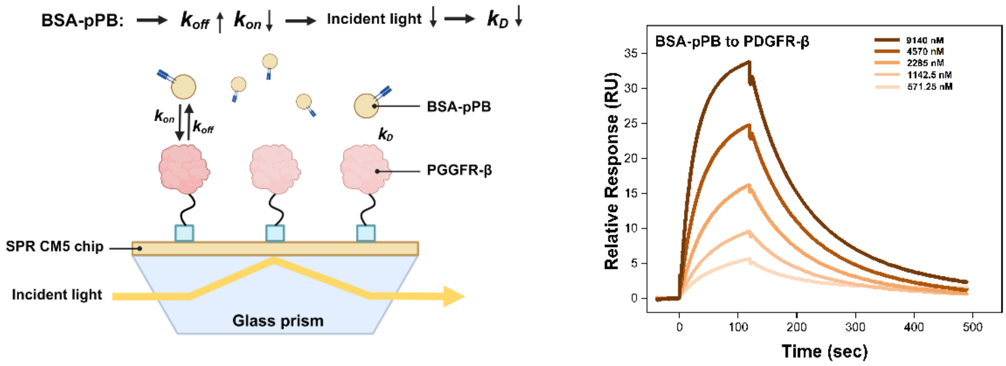

Figure S7: The SPR affinity profile of BSA-pPB with PDGFRβ.

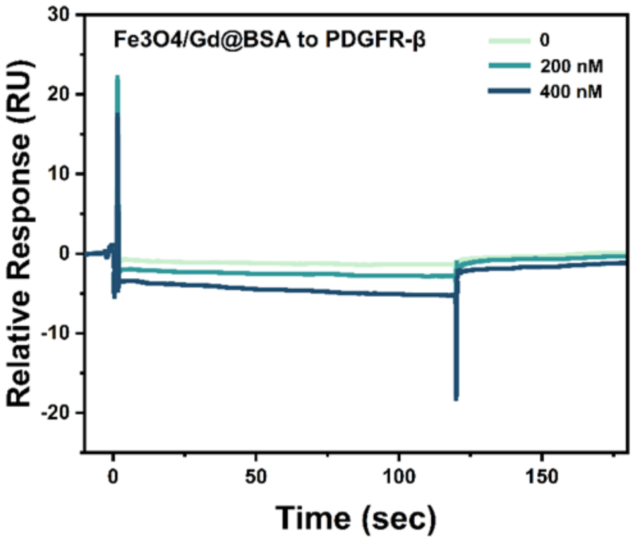

Figure S8: The affinity curves of Fe_3O_4/Gd@BSA for PDGFRβ.

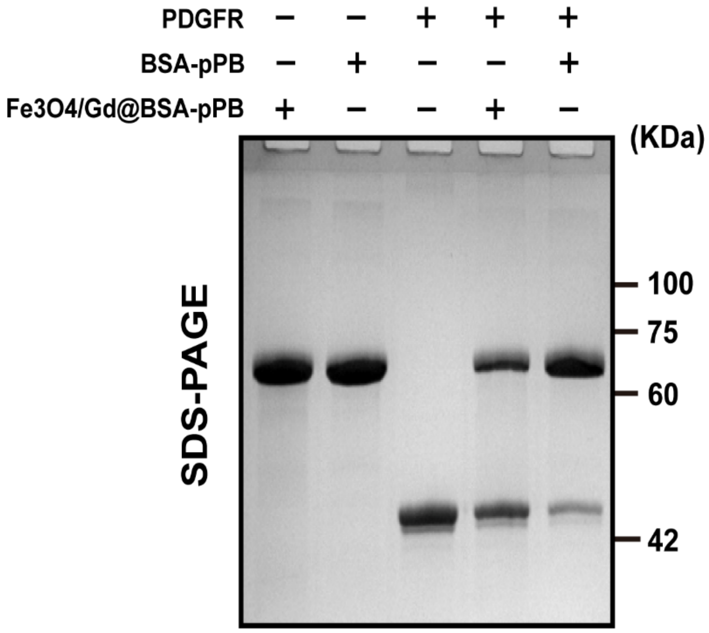

Figure S9: The SDS-PAGD analysis of PDGFR capture by Fe_3O_4/Gd@BSA-pPB probe and BSA-pPB, respectively.

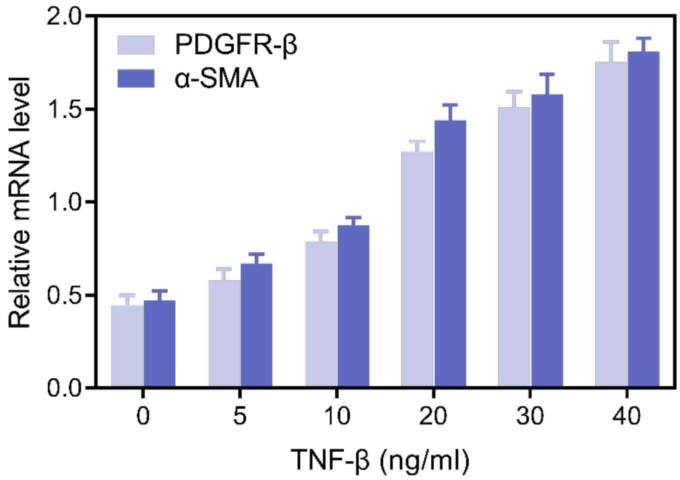

Figure S10: The mRNA level changes in the addition of different concentrations of TNF-β-activated LX-2.

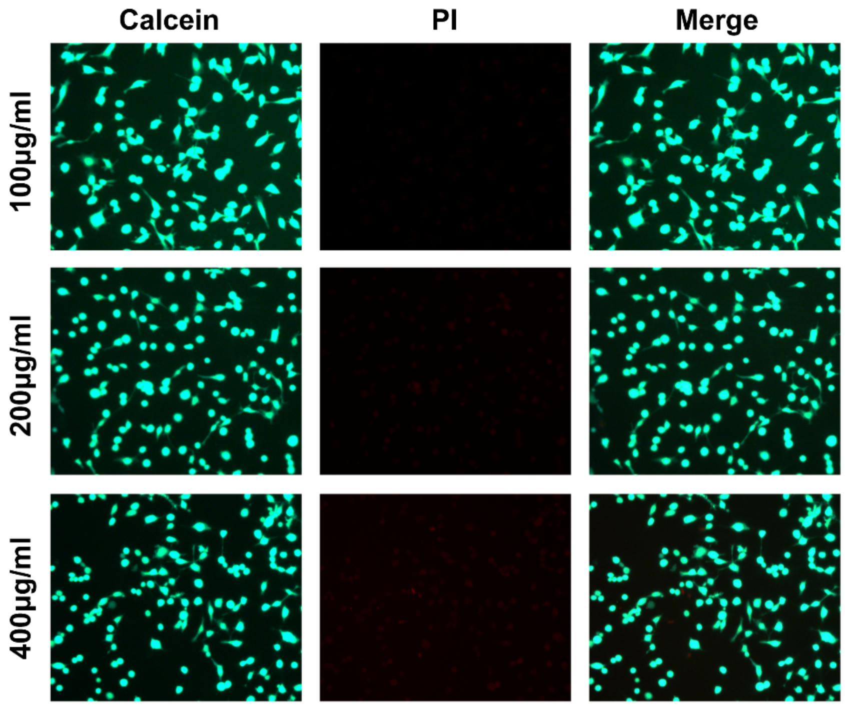

Figure S11: The live-dead staining (AM/PI) of 293T at different concentrations of Fe_3O_4/Gd@BSA-pPB.

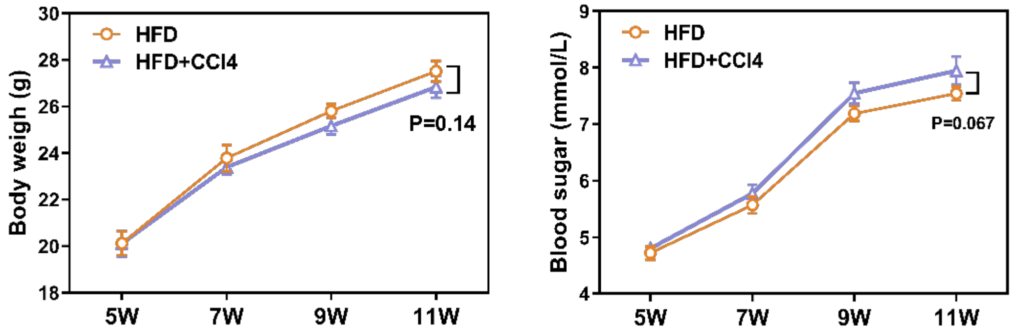

Figure S12: The body weigh and blood sugar of early liver fibrosis models for different induction times (n = 3 biologically independent samples).

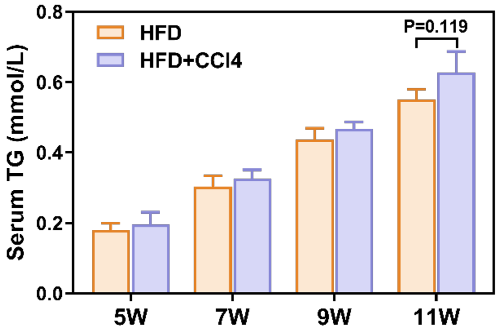

Figure S13: Serological analysis (TG) of early liver fibrosis models for different induction times (n=3).

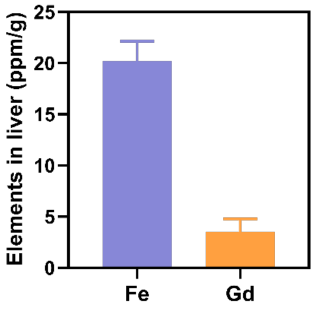

Figure S14: Elemental analysis of the liver was conducted 60 minutes post-injection of ultrasensitive Fe_3O_4/Gd@BSA-pPB probes.

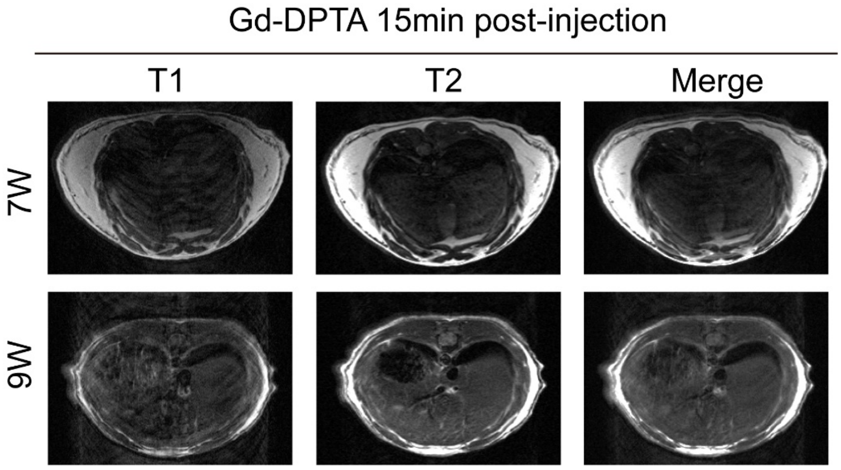

Figure S15: T1 and T2 imaging of Gd-DPTA 15 min after injection in early non-alcoholic fatty liver fibrosis.

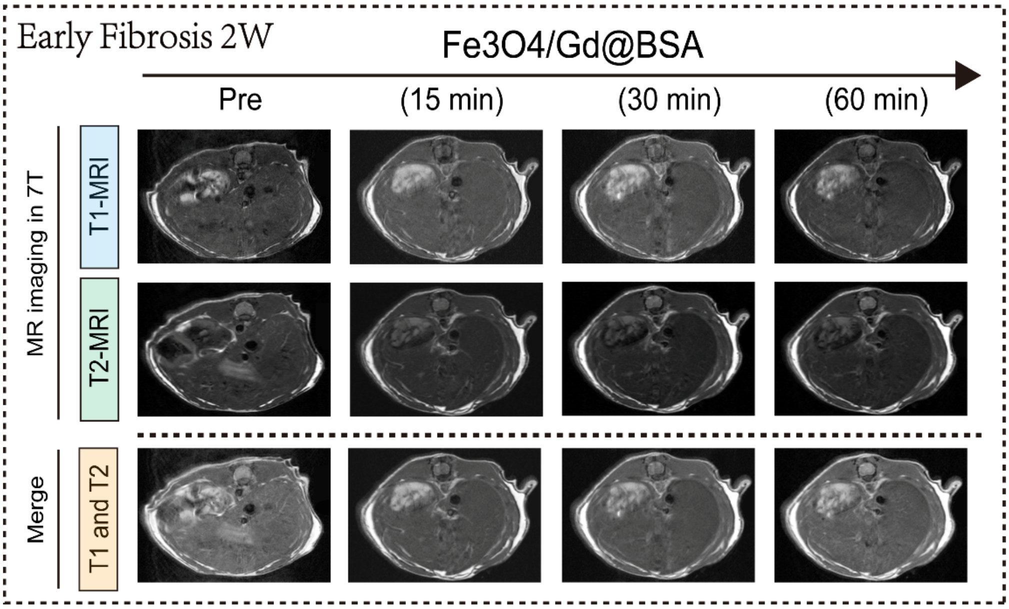

Figure S16: Imaging effect of Fe_3O_4/Gd@BSA in 2W hepatic fibrosis.

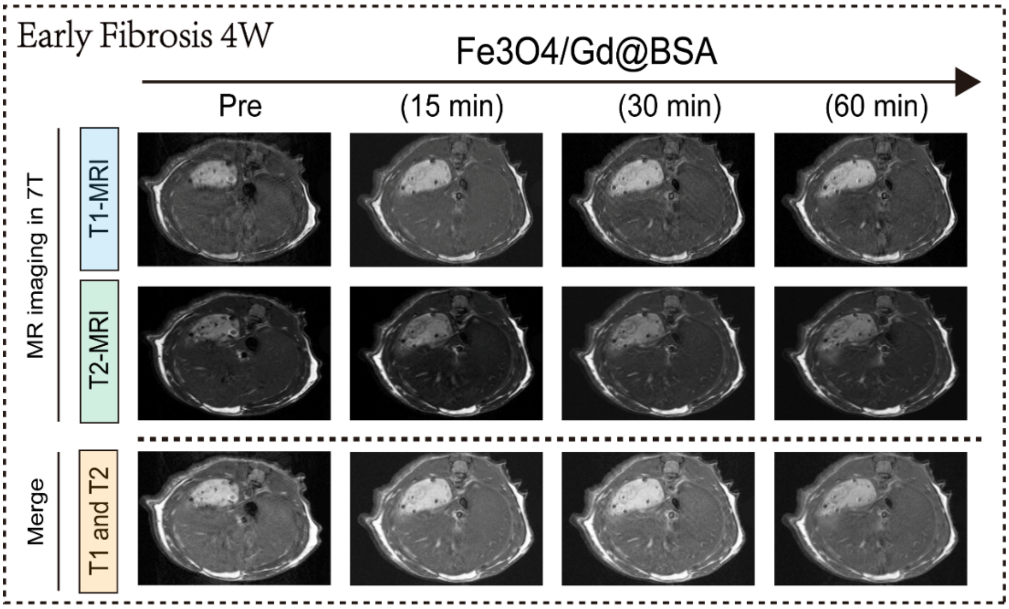

Figure S17: Imaging effect of Fe_3O_4/Gd@BSA in 4W hepatic fibrosis.

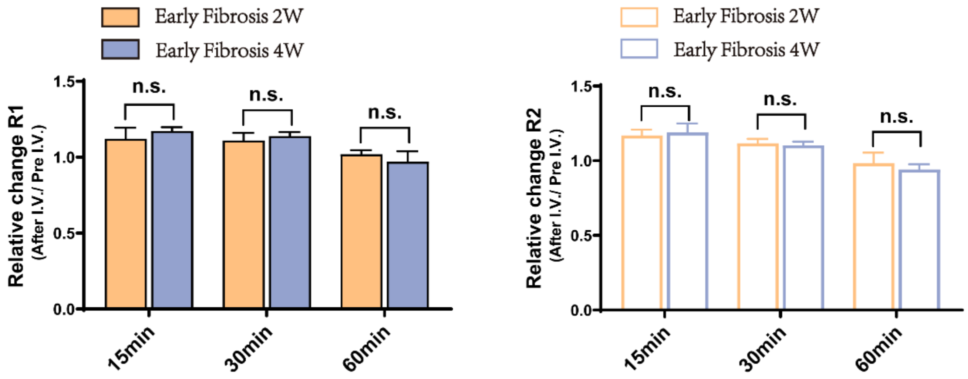

Figure S18: Relative changes in R1 and R2 after Fe_3O_4/Gd@BSA injection.

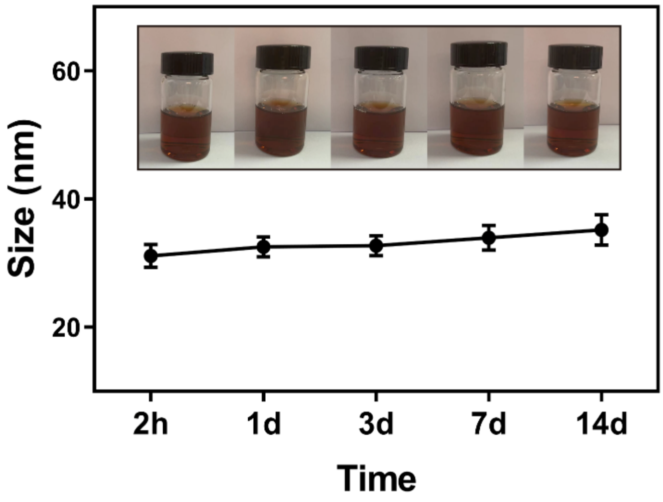

Figure S19: Hydrated particle size and corresponding photos of the ultrasensitive Fe_3O_4/Gd@BSA-pPB probes(250 μg/ml) at different times.

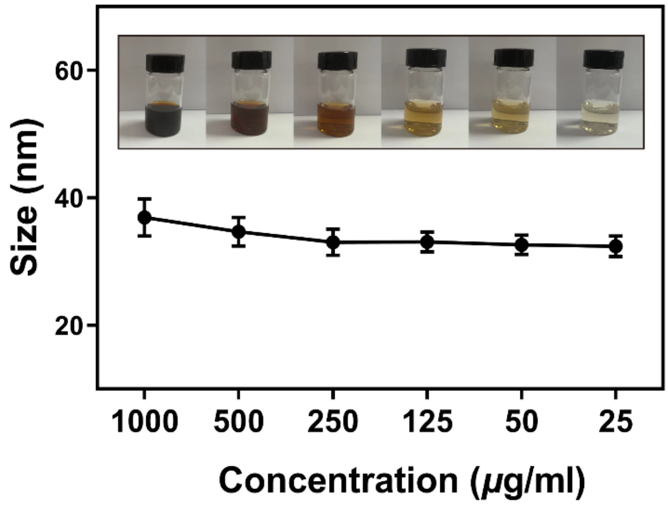

Figure S20: Hydrated particle size and corresponding photos of ultrasensitive Fe_3O_4/Gd@BSA-pPB probes at different concentrations (25-1000 μg/ml) after 48h.

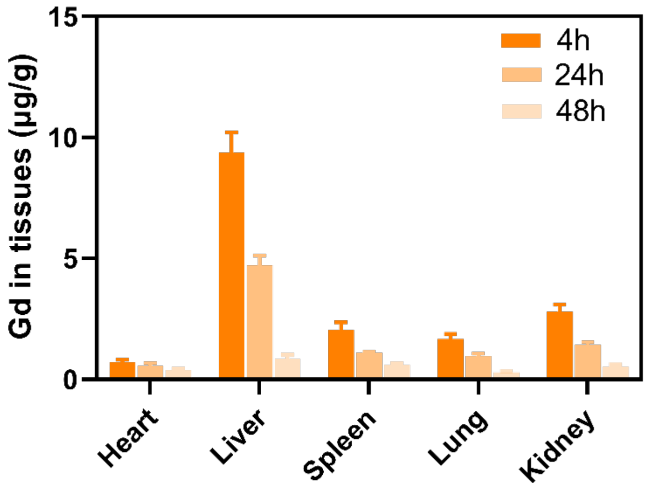

Figure S21: Time-dependent biodistribution analysis of Gd in major organs 4, 24 and 48 h after injection.

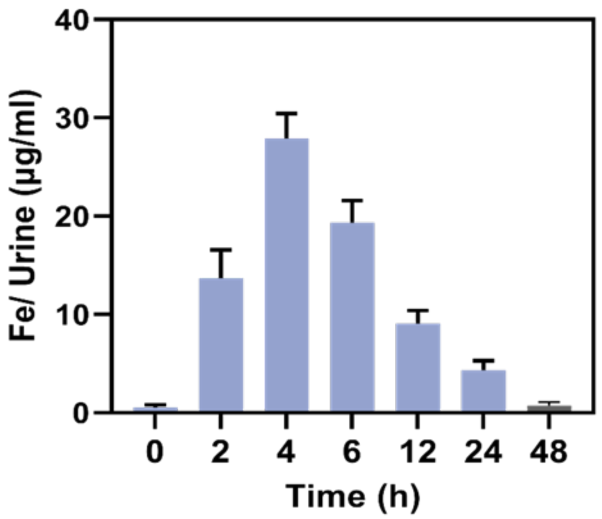

Figure S22: Urinary iron content at different times after intravenous injection of Fe3O4/Gd@BSA-pPB nanoprobe.

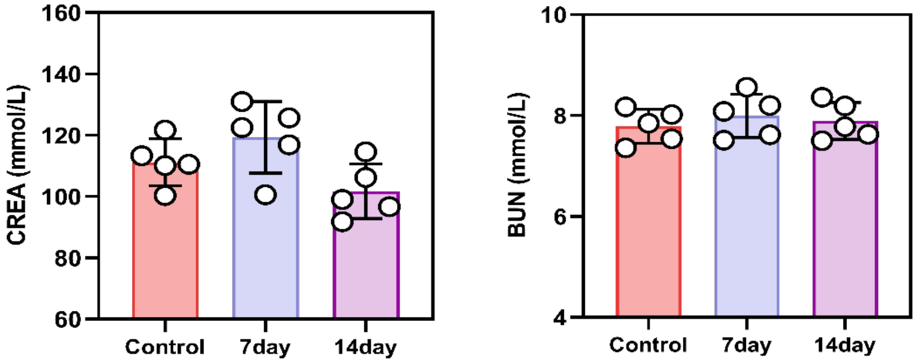

Figure S23: CREA and BUN were measured 7 and 14 days after injection

Reference:

[1] S.M. Wu, T.T. Xu, J.H. Gao, Q. Zhang, Y.X. Huang, Z.L. Liu, X.Z. Hao, Z.W. Yao, X. Hao, P.Y. Wu, Y. Wu, B. Yin, Z.M. Tang, Non-invasive diagnosis of liver fibrosis via MRI using targeted gadolinium-based nanoparticles, European Journal of Nuclear Medicine and Molecular Imaging, 52 (2024) 48-61.

[2] C. Jin, P.F. Wu, Y.S. Yang, Z.X. He, H.L. Zhu, Z. Li, A novel fluorescent probe for the detection of peroxynitrite and its application in acute liver injury model, Redox Biology, 46 (2021).

[3] P.S. Zhang, Y.Y. Qiao, L.C. Zhu, M. Qin, Q.L. Li, C. Liu, Y.P. Xu, X. Zhang, Z.H. Gan, Y. Hou, Nanoprobe Based on Biominerals in Protein Corona for Dual-Modality MR Imaging and Therapy of Tumors, Acs Nano, 17 (2023) 184-196.

[4] S.L. Friedman, V. Ratziu, S.A. Harrison, M.F. Abdelmalek, G.P. Aithal, J. Caballeria, S. Francque, G. Farrell, K.V. Kowdley, A. Craxi, K. Simon, L. Fischer, L. Melchor-Khan, J. Vest, B.L. Wiens, P. Vig, S. Seyedkazemi, Z. Goodman, V.W.S. Wong, R. Loomba, F. Tacke, A. Sanyal, E. Lefebvre, A randomized, placebo-controlled trial of cenicriviroc for treatment of nonalcoholic steatohepatitis with fibrosis, Hepatology, 67 (2018) 1754-1767.

[5] A. Omelyanchik, A.S. Kamzin, A.A. Valiullin, V.G. Semenov, S.N. Vereshchagin, M. Volochaev, A. Dubrovskiy, T. Sviridova, I. Kozenkov, E. Dolan, D. Peddis, A. Sokolov, V. Rodionova, Iron oxide nanoparticles synthesized by a glycine-modified coprecipitation method: Structure and magnetic properties, Colloids and Surfaces a-Physicochemical and Engineering Aspects, 647 (2022).
